# Octreotide Conjugates for Tumor Targeting and Imaging

**DOI:** 10.3390/pharmaceutics11050220

**Published:** 2019-05-07

**Authors:** Eduard Figueras, Ana Martins, Adina Borbély, Vadim Le Joncour, Paola Cordella, Raffaella Perego, Daniela Modena, Paolo Pagani, Simone Esposito, Giulio Auciello, Marcel Frese, Paola Gallinari, Pirjo Laakkonen, Christian Steinkühler, Norbert Sewald

**Affiliations:** 1 Department of Chemistry, Organic and Bioorganic Chemistry, Bielefeld University, Universitätsstraße 25, DE-33615 Bielefeld, Germany; eduard.figueras@uni-bielefeld.de (E.F.); ana_martins@exiris.it (A.M.); adina.borbely@uni-bielefeld.de (A.B.); marcel.frese@uni-bielefeld.de (M.F.); 2 Exiris srl, Via di Castel Romano 100, IT-00128 Rome, Italy; paola_gallinari@exiris.it (P.G.); christian_steinkuhler@exiris.it (C.S.); 3 Translational Cancer Medicine Research Program, Faculty of Medicine, Haartmaninkatu 8, University of Helsinki, FI-00014 Helsinki, Finland; vadim.lejoncour@helsinki.fi (V.L.J.); pirjo.laakkonen@helsinki.fi (P.L.); 4 Italfarmaco SpA, Via dei Lavoratori 54, IT-20092 Cinisello Balsamo, Italy; p.cordella@italfarmaco.com (P.C.); r.perego@italfarmaco.com (R.P.); d.modena@italfarmaco.com (D.M.); p.pagani@italfarmaco.com (P.P.); 5 IRBM SpA, Via Pontina km. 30,600, IT-00071 Pomezia, Italy; s.esposito@irbm.com (S.E.); g.auciello@irbm.com (G.A.)

**Keywords:** tumor targeting, small molecule drug conjugates, cryptophycin, octreotide, cytotoxic payloads, imaging

## Abstract

Tumor targeting has emerged as an advantageous approach to improving the efficacy and safety of cytotoxic agents or radiolabeled ligands that do not preferentially accumulate in the tumor tissue. The somatostatin receptors (SSTRs) belong to the G-protein-coupled receptor superfamily and they are overexpressed in many neuroendocrine tumors (NETs). SSTRs can be efficiently targeted with octreotide, a cyclic octapeptide that is derived from native somatostatin. The conjugation of cargoes to octreotide represents an attractive approach for effective tumor targeting. In this study, we conjugated octreotide to cryptophycin, which is a highly cytotoxic depsipeptide, through the protease cleavable Val-Cit dipeptide linker using two different self-immolative moieties. The biological activity was investigated in vitro and the self-immolative part largely influenced the stability of the conjugates. Replacement of cryptophycin by the infrared cyanine dye Cy5.5 was exploited to elucidate the tumor targeting properties of the conjugates in vitro and in vivo. The compound efficiently and selectively internalized in cells overexpressing SSTR2 and accumulated in xenografts for a prolonged time. Our results on the in vivo properties indicate that octreotide may serve as an efficient delivery vehicle for tumor targeting.

## 1. Introduction

Cancer therapy has experienced several paradigm changes during the last decade from small molecule drugs over targeted therapy with antibody-drug conjugates (ADCs) to approaches in immuno-oncology. Originally, tumor therapy was based on cytotoxic drugs as mono or combination therapy (alkylating agents like cisplatin, chlorambucil, procarbazine, carmustine; antimetabolites like methotrexate, cytarabine, gemcitabine; microtubule-binding agents like vinblastine, paclitaxel, or topoisomerase inhibitors). Traditional chemotherapeutics (e.g., paclitaxel, doxorubicin) usually do not preferentially accumulate in tumors, but affect all tissues, which leads to detrimental side effects [1]. Thus, targeted therapy with antibody-drug conjugates (ADCs) and, more recently, small molecule-drug conjugates (SMDCs), has emerged as a viable alternative to enlarging the therapeutic window [2,3,4,5,6]. With the approvals of Adcetris^®^ (Seattle Genetics/Millennium), Kadcyla^®^ (Genentech/Roche), Besponsa^®^ (Wyeth/Pfizer), the re-approval of Mylotarg^®^ (Wyeth/Pfizer), and more than 80 ADCs in clinical trial pipelines, ADCs are to be considered as a new class of pharmaceuticals. When compared to classical cytotoxic drugs, ADCs have the benefit of higher specificity towards tumor cells and controllable release mechanisms at the site of action. Small molecule-mediated targeting represents advantages in terms of the straightforward organic synthesis of SMDCs and their uniform structure as compared to antibodies. Indeed, small molecule ligands have been used to efficiently target tumors expressing the folate receptor [7], prostate-specific membrane antigen (PSMA) [8], carbonic anhydrase IX (CAIX) [9,10,11], and somatostatin receptors (SSTRs) [12].

The somatostatin receptors (SSTRs) belong to the G-protein-coupled receptor family and they can be subdivided into five different subtypes (SSTR1-SSTR5). Many neuroendocrine tumors (NETs) overexpress the somatostatin receptor genes, especially SSTR2, followed by SSTR1 and SSTR5 [13]. Octreotide is a short synthetic octapeptide that is derived from the natural somatostatin with high affinity and selectivity for SSTR2. Moreover, it has a better metabolic stability and half-life than somatostatin due to the downsizing of the ring and the incorporation of D-amino acids [14]. The replacement of the disulfide bridge into a dicarba-analogue can further enhance the stability [15,16,17]. As a result, octreotide and the analogues of it have been used to specifically deliver a large number of cytotoxic agents, such as paclitaxel [18], doxorubicin [19], periplocymarin [20], or periplogenin [21]. Moreover, octreotide is routinely used in clinical practice for tumor diagnostic imaging (e.g., OctreoScan, ^68^Ga-DOTATATE) [22,23].

In this regard, the use of potent cytotoxic agents is an attractive approach to increasing the efficacy and reducing the dosage. Hence, cryptophycins, which are potent microtubule destabilizers, represent promising agents to be conjugated to octreotide for tumor targeting [24].

Cryptophycins are naturally occurring cyclic depsipeptides that were first isolated from cyanobacteria [25]. They target tubulin and block the microtubule formation, leading to high cytotoxicity against many cancer cell lines. Moreover, they are a weak substrate for the P-gp efflux pump and, consequently, the cytotoxicity is only slightly reduced in multidrug-resistant (MDR) cancer cells [26]. Due to these characteristics, several cryptophycin analogues were investigated as chemotherapeutics, and Eli Lilly brought cryptophycin-52 (LY-355703) to the clinics. However, the clinical development was discontinued during phase II because of side effects and insufficient efficacy [27,28]. Subsequent research focused on several structure-activity relationship studies [29,30,31,32,33]. Special emphasis was placed on the introduction of a functional group, enabling the conjugation to a homing device for targeted tumor therapy [34,35,36,37,38,39,40].

Sanofi and Genentech developed cryptophycin derivatives as payloads in the ADC field [41,42,43]. In particular, cryptophycin that was modified in the *para* position of the phenyl ring in unit A has been used in this context, but it has shown to be highly unstable in murine plasma, which made the use of these conjugates impossible for preclinical development of new ADCs. Stability problems of the macrocycle could be subsequently overcome by applying modifications in the payload [44] or changing the anchoring point to the antibody [45]. In strong contrast, little is known regarding small molecules that are conjugated to cryptophycin as delivery agents [46].

We have recently communicated the application of acetazolamide as a homing device to deliver cryptophycin-55 glycinate to tumors overexpressing carbonic anhydrase IX. This is the first report showing the in vivo data of a SMDC comprising cryptophycin [47]. This payload has shown high potency and good stability in murine and human plasma, making it a promising agent to be used in targeted therapy [48]. Here, we describe the employment of cryptophycin-55 glycinate as a potent payload to be released in tumors overexpressing SSTR2 using octreotide as the delivery vehicle. These findings demonstrate that efficient tumor delivery can be achieved with small molecules, such as octreotide, and that cryptophycin is a valid payload for targeted tumor therapy.

## 2. Materials and Methods

### 2.1. Synthesis and Structural Characterization of Compounds **1**–**8**

**Synthesis of N_3_-PEG4-Val-Cit-Pro-Gly (1) and N_3_-PEG4-Val-Cit-Gly-Pro (2)**: Self-immolative linkers **1** and **2** were manually synthesized using standard solid-phase peptide synthesis (SPPS). First amino acid was loaded to chlorotrityl resin by reacting the corresponding amino acid (1.5 eq) and DIPEA (3 eq) in anhydrous DCM for 3 h at RT. Subsequently, MeOH (1 mL) was added, the resin was stirred for 10 min, and it was then washed with DMF (6 × 1 min) and DCM (1 × 1 min). The resin was dried with diethyl ether and Fmoc test was performed to check the loading. Sequential Fmoc removal and the coupling of the corresponding amino acid performed elongation of the sequence. The Fmoc group was removed by treating the resin with a mixture of piperidine/DMF (3:7, 2 + 10 min). The coupling of the Fmoc amino acids (4 eq) was performed by treatment with DIC (4 eq) and Oxyma (4 eq) in DMF under stirring at RT for 2 h. 15-Azido-4,7,10,13-tetraoxapentadecanoic acid (**13**, 2 eq) coupling was performed with DIC (2 eq), Oxyma (2 eq), and DIPEA (2 eq) in DMF under stirring at RT for 6 h. Final cleavage was performed with TFA/H_2_O/TIS (95:2.5:2.5) for 2 h at RT. Product was purified by reversed-phase HPLC (method P1). **1**: **HPLC-ESI-MS**: t_R_ = 5.33 min, >99% purity (λ = 220 nm), measured *m*/*z* = 702.43 (calculated 702.38 [M + H]^+^); **2**: **HPLC-ESI-MS**: t_R_ = 5.52 min, 91% purity (λ = 220 nm), measured *m*/*z* = 702.48 (calculated 702.38 [M + H]^+^).

**Synthesis of N_3_-PEG4-Val-Cit-Pro-Gly-Cry-55gly (4)**: Cryptophycin-55 glycinate trifluoroacetate prepared as previously described [47] (5 mg, 5.7 µmol, 1 eq), **1** (8 mg, 11.4 µmol, 2 eq), PyBOP (5.9 mg, 11.4 µmol, 2 eq), and HOBt∙H_2_O (2 mg; 12.8 µmol; 2.25 eq) were placed under argon atmosphere and then dissolved with anhydrous DMF (0.5 mL). DIPEA (2.5 µL, 14.3 µmol, 2.5 eq) was added and the reaction mixture was stirred at RT for 4 h. Subsequently, the solution was directly purified by RP-HPLC (method P3). Freeze-drying of fractions containing the product afforded **4** (4.5 mg, 54% yield) as white powder. **HPLC-ESI-MS**: t_R_ = 9.57 min, >99% purity (λ = 220 nm), measured *m*/*z* = 1445.69 (calculated 1445.65 [M + H]^+^); measured *m*/*z* = 723.35 (calculated 723.33 [M + 2H]^2+^). **HRMS (ESI-MS):** *m*/*z* calculated for C_67_H_100_Cl_2_N_12_O_19_ [M + 2H]^2+^ 723.3297; found 723.3291.

**Synthesis of N_3_-PEG4-Val-Cit-Gly-Pro-Cry-55gly (5)**: Cryptophycin-55 glycinate trifluoroacetate (10 mg, 0.011 mmol, 1 eq), **2** (16 mg, 0.022 mmol, 2 eq), PyBOP (11.9 mg, 0.022 mmol, 2 eq), and HOBt∙H_2_O (3.9 mg; 0.026 mmol; 2.25 eq) were placed under argon atmosphere and then dissolved with anhydrous DMF (0.5 mL). DIPEA (5 µL, 0.028 mmol, 2.5 eq) was added and the reaction mixture was stirred at RT for 4 h. Afterwards, the solution was directly purified by RP-HPLC (method P2). Freeze-drying of fractions containing the product afforded **5** (13 mg, 79% yield) as white powder. **HPLC-ESI-MS**: t_R_ = 9.54 min, 97% purity (λ = 220 nm), measured *m*/*z* = 1445.67 (calculated 1445.65 [M + H]^+^); measured *m*/*z* = 723.34 (calculated 723.33 [M + 2H]^2+^). **HRMS (ESI-MS):** *m*/*z* calculated for C_67_H_100_Cl_2_N_12_O_19_ [M + 2H]^2+^ 723.3297; found 723.3291.

**Synthesis of 4-pentynoyl-octreotide (α-CH_2_CH_2_C≡CH) (6)**: Octreotide acetate was ε-mono-Boc-protected, as previously described [49]. Octreotide(ε-Boc) (69 mg, 0.056 mmol, 1 eq), was dissolved in a 1:1 solution of ethanol and 0.2 M borate buffer (pH = 8.5) to a final concentration of 2 mg/mL. Subsequently, 4-pentynoic acid succinimidyl ester (109 mg, 0.56 mmol, 10 eq) was added and the solution was stirred at RT overnight. The crude was purified by RP-HPLC (method P1) to obtain Octreotide (ε-Boc, α-CH_2_CH_2_C≡CH) (53 mg, 80% yield). Finally, the Boc group was removed with TFA/H_2_O/TIS (95:2.5:2.5) for 5 min at RT, the solvents were immediately evaporated, and the crude residue was purified by RP-HPLC (method P1) to give **6** (46 mg, 85% yield) as a white powder after freeze-drying. **HPLC-ESI-MS**: t_R_ = 5.97 min, 96% purity (λ = 220 nm), measured *m*/*z* = 1099.47 (calculated 1099.47 [M + H]^+^); measured *m*/*z* = 550.24 (calculated 550.24 [M + 2H]^2+^). **HRMS (ESI-MS):** *m*/*z* calculated for C_54_H_71_N_10_O_10_S_2_ [M + H]^+^ 1099.4740; found 1099.4775; calculated for C_54_H_72_N_10_O_10_S_2_ [M + 2H]^2+^ 550.2406; found 550.2429.

**Synthesis of 7**: Azide **4** (6 mg, 0.004 mmol, 1 eq), alkyne **6** (5 mg, 0.004 mmol, 1 eq), CuSO_4_·5H_2_O (0.6 mg, 0.6 eq), and sodium ascorbate (0.3 mg, 0.4 eq) were placed under argon atmosphere and dissolved with a degassed solution of DMF/H_2_O (1:1, 0.5 mL). The solution was stirred for 24 h at 40°C and it was directly purified by RP-HPLC (method P4). Freeze-drying of desired fractions afforded **7** (5.6 mg, 51% yield) as white powder. **HPLC-ESI-MS**: t_R_ = 7.47 min, 98% purity (λ = 220 nm), measured *m*/*z* = 1272.62 (calculated 1272.56 [M + 2H]^2+^); measured *m*/*z* = 848.75 (calculated 848.71 [M+3H]^3+^). **HRMS (ESI-MS):** *m*/*z* calculated for C_121_H_171_Cl_2_N_22_O_30_S_2_ [M+3H]^3+^ 848.7111; found 848.7122.

**Synthesis of 8**: Azide **5** (10.8 mg, 0.007 mmol, 1 eq), alkyne **6** (8.5 mg, 0.007 mmol, 1 eq), CuSO_4_·5H_2_O (1.1 mg, 0.6 eq), and sodium ascorbate (0.6 mg, 0.4 eq) were placed under argon atmosphere and dissolved with a degassed solution of DMF/H_2_O (1:1, 0.5 mL). The solution was stirred for 24 h at 40 °C and it was directly purified by RP-HPLC (method P2). Freeze-drying of desired fractions afforded **8** (12 mg, 60% yield) as white powder. **HPLC-ESI-MS**: t_R_ = 7.50 min, 98% purity (λ = 220 nm), measured *m*/*z* = 1272.58 (calculated 1272.56 [M + 2H]^2+^); measured *m*/*z* = 848.72 (calculated 848.71 [M+3H]^3+^). **HRMS (ESI-MS):** *m*/*z* calculated for C_121_H_171_Cl_2_N_22_O_30_S_2_ [M+3H]^3+^ 848.7111; found 848.7139.

### 2.2. Biological Evaluation

**Binding affinity**: The compounds were dissolved in 100% DMSO at a concentration of 10 mM and diluted with assay buffer (25 mM HEPES pH 7.4, 5 mM MgCl_2_, 1 mM CaCl_2_, 10 µg/mL Saponin, 0.5% protease free BSA). The dilution of test compound (50 µL, maximum final DMSO concentration 3%), radioligand 3-[125I] iodotyrosyl11 Somatostatin 14 (Perkin Elmer, NEX389, 25 µL, 0.4 nM), and membrane extract (recombinant CHO-K1-SST2 (NP_001041.1), 25 µL, 0.2 µg) were successively added to a 96-well plate and then incubated at 25 °C for 60 min. It was filtered over GF/B Unifilter plate (Perkin Elmer, 6005177, pre-soaked in 0.5% PEI for 2 h at room temperature) with a Filtermate Harvester (Perkin Elmer). After rinsing the filters six times with 0.5 mL of ice-cold washing buffer (25 mM HEPES pH 7.4, 5 mM MgCl_2_, 1 mM CaCl_2_), 50 µL of Microscint 20 (Packard) was added to the filters and the samples were incubated 15 min on an orbital shaker and then counted with a TopCount™ for 1 min/well. The binding affinity was determined in duplicates by plotting the dose-response data to a nonlinear regression with variable slope.

**In vitro cytotoxicity**: To evaluate the cytotoxicity of conjugates and metabolites, AtT20/D16v-F2 murine pituitary tumor cells obtained from American Type Culture Collection (ATCC, Bethesda, MD, USA) were used. Cell viability was determined using the MTT (3-(4,5-dimethyl thiaz ol-2-yl)-2,5-diphenyltetrazolium bromide) assay. Briefly, cells in DMEM supplemented with 10% FBS, 1% L-glutamine, and 1% penicillin/streptomycin were seeded in 96-well culture plates (3000 cells/well) and incubated overnight in a humidified, 37 °C, 5% CO_2_ atmosphere to allow for adherence. The following day, cells were treated with serial dilutions of each compound starting at 100 nM for free drugs and 1000 nM for each one of the conjugates and incubated as described for 2 h. 0.1% DMSO served as a control. After incubation, the cells were washed once, media replaced, and incubation continued for further 70 h. At the end of treatment, 5 µL of MTT solution (5 mg/mL in deionized H_2_O, Sigma #M5655) was added to each well and the cells were incubated for another 2 h. Finally, 100 µL of lysis buffer (10% SDS, 10 mM HCl) was added, and the cells were placed in the incubator overnight for the formazan crystal solubilization. Absorbance at 540 nm was measured using a FLUOstar Omega (BMG Labtech) microplate reader and the growth inhibition ratio was calculated. Blank controls detecting cell-free media absorbance were performed in parallel. Three experimental replicates were used. The half-maximal inhibitory concentration values (IC_50_) were obtained from viability curves while using GraphPad Prism 6. Cell viability was expressed as percentage relative to the respective control conditions.

**Plasma stability**: The sample preparation and the metabolic analysis of the conjugates was carried out similarly to the previously described method [50]. Shortly, in a 96-well plate each conjugate and the control compound, procaine (3 mM, stock solution in DMSO) in three replicates were diluted with 250 μL of plasma to give a 3 µM concentration and then incubated at 37 °C. At each sample collecting time point, an aliquot of 30 µL was transferred into a 96-deep well plate and the reaction was stopped with 120 µL of acetonitrile containing 0.1% formic acid and 0.4 µg/mL of warfarin as the internal standard (IS). This mixture was centrifuged at 1100 ×g for 30 min at 4 °C and 50 µL of supernatant was transferred into a clean 96 deep-well plate and diluted with 50 µL of 0.1% formic acid in water. The samples were stored at -80 °C until analyses. Stability was determined based on LC-HRMS analysis of the disappearance of the compound as a function of incubation time, while using area ratio (analyte peak area vs internal standard peak area). The elimination constant k is calculated by plotting mean disappearance values on a semi-logarithmic scale and fitting with a best fit linear regression. The half-life (*t*_1/2_) expressed in hours is derived using the equation: *t*_1/2_ = ln2/(−k).

**Receptor and conjugate internalization**: For the immunofluorescence studies of SSTR2 internalization, the AtT20/D16v-F2 cells were grown overnight in eight-well chamber slides (Nunc^®^ Lab-Tek^®^, Sigma-Aldrich, St. Louis, MO, USA) previously coated with poly-D-lysine (10 µg/mL, Sigma-Aldrich, St. Louis, MO, USA). The cells were treated with concentrations that ranged from 10 to 1000 nM of either octreotide-cryptophycin conjugates or with octreotide as positive control, for 30 min at 37 °C. To stop the internalization process and fix the cells, the wells were rinsed twice with cold PBS and fixed with ice cold methanol for 7 min. Subsequently, the non-specific binding sites were blocked with PBS containing 10% fetal bovine serum for 60 min at room temperature, and the cells were incubated for another 60 min with rabbit anti-SSTR2 primary antibody (UMB-1, Abcam #ab134152) diluted 1:200 in blocking solution. Next, the wells were rinsed three times for 5 min with PBS followed by incubation in the dark with the secondary antibody Alexa Fluor 488 goat anti-rabbit diluted 1:600 in blocking solution for 60 min. Nuclei were visualized by DAPI staining (1 µL/mL in PBS, Tocris) for 15 min. Finally, the wells were rinsed, chambers removed, and coverslips mounted with Mowiol 4-88 antifading solution (Sigma). Images were generated using the Zeiss AxioImager upright epifluorescence microscope, with a 100× oil immersion objective. The real-time internalization of the labeled conjugate **10** (synthesis described in the Supplementary Material) was acquired using a Zeiss LSM880 confocal microscope coupled to an environmental chamber, allowing a 37 °C, 5% CO_2_ atmosphere. Briefly, the AtT20/D16v-F2 murine pituitary tumor cells that express high levels of SSTR2 and the human epithelial lung carcinoma A549 cells with low SSTR2 expression were grown overnight, as described above. On the day of the experiment, either 1 µM of the fluorescently labeled conjugate **10** alone or a mixture of that with 100-fold excess of free octreotide were prepared in phenol red-free media (1% DMSO final) and warmed up to 37 °C. Image acquisition was performed using a 40× water immersion objective and it started shortly after the solutions were added to the cells. One frame was acquired every 30 s for a total of 60 frames. Brightfield was used to visualize the cellular bodies.

**Animal studies:** Animal experiments were approved by the Committee for Animal Experiments of the District of Southern Finland (ESAVI/6285/04.10.07/2014).

**Pharmacokinetics:** To evaluate the pharmacokinetic profile of conjugate **8** and its fluorescence-labeled analogue **10**, four-weeks-old female heterozygous NCR mice from Charles River were used. Animals were intravenously injected with 2.5 mg/kg (in 2% DMSO in water, dosed at 0.5 mg/mL) of each conjugate. Terminal blood samples were collected via cardiac puncture from animals under deep anesthesia at different time points (5, 15, 30, 60, 180, and 360 min, *n* = 3). The samples were rapidly transferred to lithium heparin-coated tubes, kept on ice, and subsequently centrifuged at 2000 ×*g*, 4 °C for 10 min. After centrifugation, plasma was collected and the samples were kept at -20 °C until analyses.

**Tumor targeting studies:** The fluorescence-labeled conjugate **10** (1 mg/kg in 2% DMSO in water) was intravenously injected in nude mice bearing AtT20 tumors. At each time point, whole body imaging was acquired using the Lago optical imaging system (Spectral Instruments Imaging), while mice were under isoflurane anesthesia (2.5%/20% O_2_).

**Cathepsin B degradation**: An aqueous solution of L-cysteine (0.28 M) was diluted 1:10 in acetate buffer/EDTA 1 mM pH 5.5, to achieve a working solution of 28 mM of L-cysteine. This solution was used to dilute cathepsin B from bovine spleen (Sigma Aldrich, C6286) to 1.11 U/mL. The cathepsin B solution was pre-incubated at 37 °C for 15 min and then split into 45 µL aliquots in Eppendorf tubes, with two replicates for each incubation time. 5 µL of substrate solution (50 µM in MeOH/H_2_O (1:1)) were added to each tube for a final concentration of 5 µM. Tubes corresponding to t_0_ contained 100 µL of 1% HCOOH in MeOH and they were put in an ice bath to inhibit the reaction. All of the other tubes were incubated at 37 °C in an oscillating thermostatic bath and the reaction was stopped at the following incubation times: 0.25, 0.5, 1, 2, 4, and 6 h, as described for the t_0_ samples. The samples were then centrifuged for 10 min at 14000 rpm at 4 °C and filtered through regenerated cellulose syringe filters prior to injection in the HPLC-ESI-MS system. Control solutions containing 5 µM substrate in acetate buffer pH 5.5/EDTA 1 mM/L-cysteine 28 mM were also prepared and then incubated up to 6 h at 37 °C in the absence of the enzyme to check the substrate stability under incubation conditions. Substrates and their possible cleavage products (Gly-Pro-Cry-55gly, Cry-55gly, and Cry-55) were quantified via calibration curves that were prepared in acetate buffer/EDTA/L-cysteine in the range 25–5000 nM.

## 3. Results and Discussion

### 3.1. Design and Synthesis of Octreotide-cryptophycin Conjugates

Cryptophycin-55 glycinate was selected as the payload and octreotide as the targeting moiety to develop cryptophycin conjugates that targeted SSTR2. The payload was connected to the *C*-terminus of the cathepsin B-cleavable dipeptide sequence Val-Cit across two different self-immolative dipeptides (Gly-Pro or Pro-Gly) that were designed to form a diketopiperazine (DKP) [51]. Since ester bonds present a notable liability in circulation, we decided to connect the self-immolative moiety to the payload via an amide bond. Peptide bonds have also been reported to be susceptible to DKP formation [52,53]. Moreover, it is also known from the ADC field that peptide linkers can be enzymatically degraded, which provides another route for efficient release of the cytotoxic agent. The *N*-terminus of the linker contained an azide moiety for subsequent copper(I)-catalyzed alkyne-azide cycloaddition (CuAAC) to an alkyne functionalized octreotide.

**Scheme 1:**
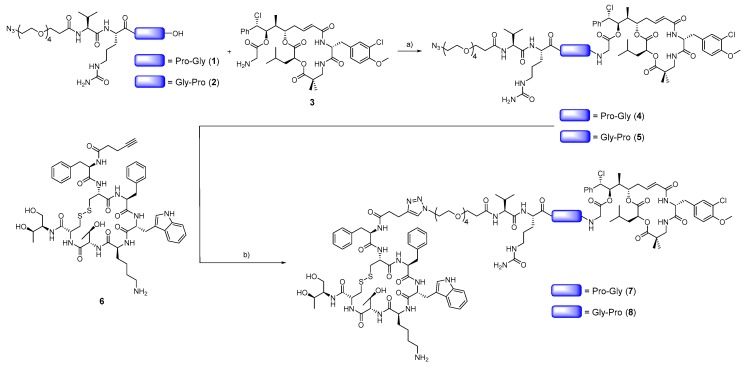
Synthesis of octreotide-cryptophycin conjugates **7** and **8**. Reagents and conditions: a) **1** or **2** (2 eq), PyBOP (2 eq), HOBt·H_2_O (2.25 eq), DIPEA (2.5 eq), DMF, RT, 4 h; b) CuSO_4_·5H_2_O (0.6 eq), sodium ascorbate (0.4 eq), DMF/H_2_O (1:1), 40 °C, 24 h.

The conjugate synthesis started with the preparation of the linkers (**1** and **2**) by using solid-phase peptide synthesis (SPPS), followed by coupling to cryptophycin-55 glycinate (**3**) across an amide bond (Scheme 1) to obtain compounds **4** and **5** in excellent purities and good yields (54–79%) after RP-HPLC purification. The final CuAAC reaction of compounds **4** and **5** with alkyne functionalized octreotide **6** resulted in the conjugates **7** and **8**. The reaction was carried out using a slight excess of CuSO_4_ in comparison to sodium ascorbate to reduce the side reactions. The reaction was gently warmed to 40 °C in order to increase the solubility of the system. Under these conditions, compounds **7** and **8** were obtained in excellent purities and satisfactory yields after RP-HPLC purification.

### 3.2. Binding Affinity

**Figure 1:**
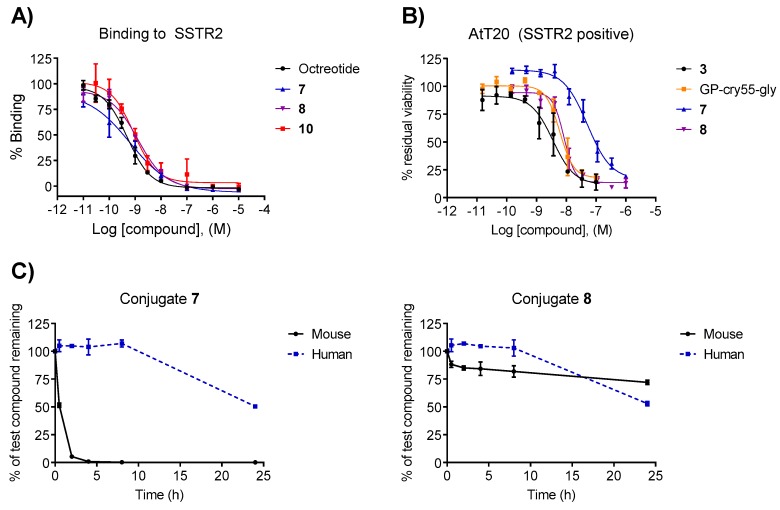
(**A**) Binding affinity towards SSTR2 of free ligand (octreotide), conjugates **7**, **8**, and **10**. (**B**) in vitro cytotoxicity of the compounds **3**, **7**, and **8** and metabolite Gly-Pro-Cry-55gly in the AtT20 cell line. (**C**) Plasma stability of conjugates **7** and **8** in murine and human plasma.

The binding affinity of the conjugates was evaluated in vitro while using a radioligand binding competition assay with human SSTR2 and it was compared to octreotide. Conjugates **7** and **8** displayed low nanomolar IC_50_ affinities (0.6 nM and 1.3 nM, respectively) that were comparable to the value of the free ligand octreotide (0.5 nM) (Figure 1A and Table 1). These results indicate that, despite the steric bulk that was introduced by the linker-payload moiety, the affinity of the conjugates remains in the low nanomolar range and the *N*-terminus of octreotide can be used for conjugation, as it is not essential for binding.

### 3.3. In Vitro Cytotoxicity

AtT20 murine pituitary cancer cells were incubated with increasing concentrations of compounds **7** and **8** to determine their cytotoxicity, which was compared to free payload **3** (Figure 1B). Cryptophycin-55 glycinate showed high potency, with an IC_50_ in the low nanomolar range (3.53 nM). Unexpectedly, the activity of conjugates **7** and **8** was remarkably different. While conjugate **8** maintained the high cytotoxicity (IC_50_ of 8.37 nM), compound **7** showed a reduced cytotoxicity (IC_50_ = 51.23 nM), which was albeit still in the nanomolar range. The reduced cytotoxicity of **7** could be attributed to the challenging and less efficient cleavage of proteases to Xaa-Pro bonds.

### 3.4. Plasma Stability

**Figure 2:**
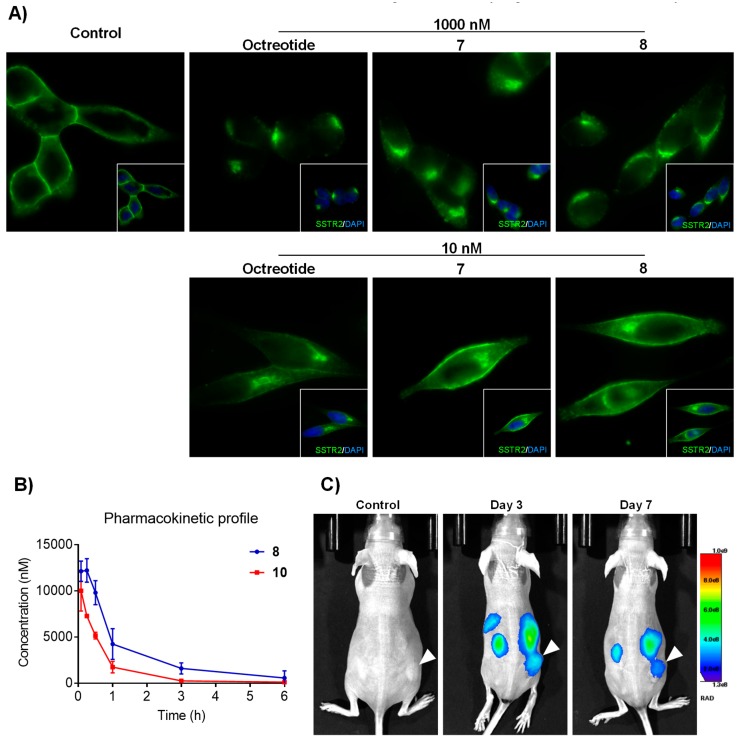
(**A**) Concentration-dependent internalization of SSTR2 in AtT20 cells. Cells were treated for 30 min at 37 °C with 10 or 1000 nM of octreotide and corresponding octreotide-cryptophycin conjugates **7** and **8**. (**B**) Pharmacokinetic profile of conjugates **8** and **10** in heterozygous NCR mice. (**C**) In vivo tumor targeting of the conjugate **10** using the AtT20 xenograft-bearing mice. Animals were intravenously injected with 1 mg/kg of conjugate **10** and whole-body images were taken at the indicated timepoints.

The stability of conjugates **7** and **8** was evaluated in vitro at 37 °C in murine and human plasma. The self-immolative moiety dramatically influenced the stability (Figure 1C). Compound **7** exhibited poor stability in mouse plasma (*t*_1/2_ = 30 min) and moderate stability in human plasma (*t*_1/2_ = 23 h). Conversely, conjugate **8** was remarkably stable in mouse plasma (*t*_1/2_ > 24 h) and also slightly more stable in human plasma compared to compound **7** (*t*_1/2_ = 24 h). The sufficient stability of compound **8** as compared to the circulatory half-life (104 min, Figure 2B) close to that measured for octreotide acetate in humans justified further investigation of its biological activity.

### 3.5. Receptor and Conjugate Internalization

In the absence of somatostatin, SSTR2 primarily localizes at the plasma membrane and it is known to be rapidly internalized upon ligand binding. However, the rate and extent of internalization can vary widely, depending on the agonist and its binding affinity [54]. When evaluating the ability to induce the internalization of the receptor in a SSTR2-positive cell model, both of the conjugates induced an internalization of SSTR2 (Figure 2A). The more stable conjugate **8** was selected and the cytotoxic moiety was replaced by the infrared dye Cy5.5 for the visualization of the conjugate to investigate the tumor homing properties of octreotide conjugates in vivo and to confirm that they internalized into the cells [55]. The dye was coupled to the spacer **2** across an amide bond (Scheme S1) to obtain the intermediate **9** that was then “clicked” to **6** using CuAAc to obtain the fluorescently labeled conjugate **10** with satisfactory yield and excellent purity (synthesis of **9** and **10** described in the SI). We obtained low nanomolar IC_50_ when the affinity of compound **10** towards SSTR2 was measured while using radioligand displacement. This confirmed that the introduction of the fluorescent dye did not alter the affinity compared to the free ligand (Figure 1A). SSTR2 expressing AtT20 cells showed effective internalization of the construct after only 10 min in live cell confocal microscopy analyses. In addition, a high concentration of the conjugate could be detected at the perinuclear space, similar to the receptor itself, upon 30 min of incubation (Figure S1 and Video S1). The internalization could be efficiently competed with a 100-fold excess of the free ligand. Moreover, no internalization was observed in A549 cells that express low levels of SSTR2, suggesting that the obtained octreotide conjugate is possibly internalized via receptor mediated endocytosis and no passive diffusion is involved in its uptake.

### 3.6. In Vivo Tumor Imaging

The tumor-targeting ability of conjugate **10** was evaluated in mice bearing AtT20 xenografts that express high levels of SSTR2. Compound **10** was intravenously administered at 1 mg/kg and whole-body imaging at different time points was performed (Figure 2C). A high percentage of the conjugate could be detected in liver and kidneys at early time points, similar to the clearance pattern of the radiolabeled octreotide derivatives [56]. However, the preferential accumulation of the compound **10** in the tumor over the healthy tissues that are not involved in the compound excretion could be observed and the conjugate showed good homing properties to tumors of small size (around 50 mm^3^) and detection up to seven days. The physicochemical properties of **10** can be significantly different when compared to **8** as the nature of the infrared dye Cy5.5 remarkably differs from cryptophycin. For this reason, it is difficult to conclude on the tumor targeting properties of compound **10** to **8**. The pharmacokinetic profile of both compounds can help to understand their behavior in vivo and the clearance properties of each. As shown in Figure 2B, conjugates **8** and **10** display similar pharmacokinetics in heterozygous mice. Indeed, conjugate **8** shows a higher area under the curve (AUC) than **10**, which is an advantage when studying its antitumor efficacy.

### 3.7. In Vivo Antitumor Activity

On the basis of the above results, the in vivo antitumor efficacy of conjugate **8** was evaluated in AtT20 tumor-bearing mice. Dose escalation experiments determined the maximum tolerated dose of the conjugate. The compound was well tolerated up to 10 mg/kg, the highest tested dose (data not shown). In comparison to the study of cryptophycin-55 glycinate as a stand-alone agent, we reduced the dose and the regimen schedule because conjugate **8** is able to selectively target the tumor site, which is not the case with the unconjugated cryptophycin. For this reason, mice were treated with compound **8** (5 mg/kg) once weekly for three weeks and the tumor volume was compared to groups that were treated with vehicle (2% DMSO in water) or a mixture of unconjugated cryptophycin and octreotide (Figure S2). However, no therapeutic benefit could be observed upon the administration of **8**. The lack of activity is presumably attributed to insufficient payload release (*vide infra*). Al though the cathepsin B sensitive linker is rapidly degraded, it is possible that the lack of self-immolation hampers an antitumor efficacy. It is usually conceived that higher and more diverse proteases are present in an in vivo system when compared to in vitro. However, it is possible that proteases that are responsible for the enzymatically degradation of the metabolite and the release of cryptophycin-55 glycinate, leading to high cytotoxicity in vitro, are present in lower amounts in nude mice bearing tumors. The activity of the conjugate and released metabolite could be significantly improved in the case of compounds with longer residence in vivo.

### 3.8. Cathepsin B Stability

**Figure 3:**
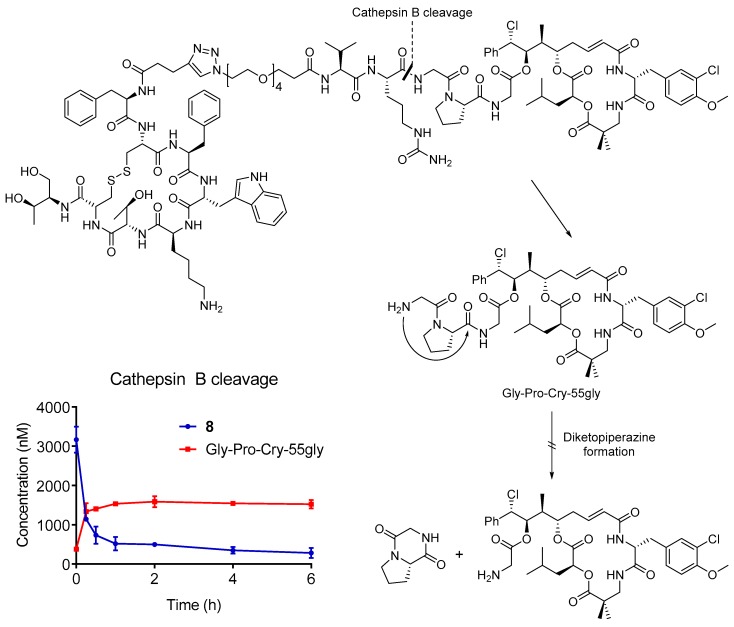
Cathepsin B cleavage and self-immolation mechanism and in vitro stability of conjugate **8** in the presence of cathepsin B and quantification of the main metabolite. Compound **8** (5 µM) was rapidly cleaved in the presence of the enzyme (1 U/mL) already at t_0_, releasing the drug containing metabolite Gly-Pro-Cry-55gly.

The cleavage of the conjugate **8** by cathepsin B was studied to elucidate the reasons for the lack of in vivo efficacy (Figure 3). The compound was highly stable in the buffer that was used for the experiment in the absence of enzyme (90% remaining after 6 h of incubation), but it was readily cleaved upon the addition of cathepsin B (1 U/mL). A small amount of compound was already metabolized at t = 0, which suggested a high reactivity of the enzyme towards conjugate **8**, which was confirmed by the fast decrease of the substrate during the first hour of incubation. As expected, the enzymatic cleavage led to the formation of Gly-Pro-Cry-55gly. However, the release of this metabolite was not followed by self-immolation of the dipeptide, as no cryptophycin-55 glycinate (**3**) was found. Further qualitative analysis did not show the presence of other metabolites (e.g., cryptophycin-55, cryptophycin-52). The cytotoxicity of the metabolite was evaluated using the same protocol than the conjugate and free drug in AtT20 cells. The IC_50_ value of the metabolite was maintained in the low nanomolar range (6.05 nM) and it was comparable to the free payload **3** and conjugate **8**.

## 4. Conclusions

Here, we validate octreotide as an appropriate delivery vehicle to tumors overexpressing SSTR2 and cryptophycin-55 glycinate as payload. The generated conjugates with cryptophycin showed good binding affinity to the receptor. The dipeptide spacer between the enzyme-labile moiety and the drug largely influenced the metabolism and cytotoxicity. While the conjugate containing the dipeptide Pro-Gly (**7**) showed instability in plasma and reduced cytotoxicity compared to the free drug, the product with the Gly-Pro sequence (**8**) was highly stable and its cytotoxicity was comparable to the unconjugated payload. The replacement of the payload for the infrared fluorophor Cy5.5 did not alter the binding properties and the labeled compound was selectively internalized in the AtT20 cells. The homing properties of **10** were studied in nude mice bearing AtT20 tumors. Pharmacokinetic and in vivo imaging studies showed that a fluorescent signal becomes rapidly associated with the tumor and persists there for many days, despite the very rapid clearance of the conjugate from circulation, upon i.v. injection. This observation points to a fundamental difference of the peptide conjugate with respect to antibody-drug conjugates. While the latter have long plasma half-lives and only slowly penetrate into tumor tissues, peptide conjugates are small enough to rapidly reach their intra-tumoral targets, where they can be trapped due to specific interaction with the cognate receptor. Glomerular filtration mostly rapidly clears the unbound conjugate, thereby avoiding any unwanted toxicities to normal tissues. ADCs, in contrast, will expose the whole organism to released toxin for a long period of time, thereby potentially giving rise to side effects. In line with this notion, several ADCs that are directed against solid tumor targets have recently failed in the clinic due to low therapeutic indices. Our octreotide-cryptophycin conjugate, albeit being well tolerated and having the desired pharmacokinetic profile, did not show any therapeutic benefit upon administration at the selected dosing regimen. Cathepsin B cleavage studies proved the efficient enzymatic cleavage of the sensitive dipeptide Val-Cit and the release of the metabolite Gly-Pro-Cry-55gly, which did not detectably react by further self-immolation to release cryptophycin-55 glycinate under the test conditions. It is possible that the in vitro cytotoxicity of the obtained metabolite is insufficient to lead to a sustained in vivo efficacy. The translation of the promising in vitro results to an in vivo model is currently under investigation. The system might benefit from a different linker, which affords the efficient release of the free payload and longer residence in vivo. The usage of different linkers and their impact on the in vivo antitumor activity are currently being studied.

## Figures and Tables

**Table 1:** Binding affinities, cytotoxicity, and plasma stability of cryptophycin-55 glycinate (**3**), metabolite GP-Cry-55 gly, and conjugates **7**, **8**, and **10** (n.d.: not determined).

Entry	Compound	Binding Affinity (IC_50_ in nM)	Cytotoxicity in AtT20 (IC_50_ in nM)	Plasma Stability (*t*_1/2_ in h)	
				Murine	Human
1	Cry-55 gly (**3**)	n.d.	3.53	> 24 [48]	> 24 [48]
2	Oct-PG-Cry (**7**)	0.6	51.23	0.5	23
3	Oct-GP-Cry (**8**)	1.3	8.37	> 24	24
4	GP-Cry-55 gly	n.d.	6.05	n.d.	n.d.
5	Oct-GP-Cy5.5 (**10**)	0.9	n.d.	n.d.	n.d.

## Supplementary Materials

The following are available online at https://www.mdpi.com/1999-4923/11/5/220/s1: General methods and instrumentation, synthesis of compounds **9**-**13**, characterization of intermediates and final products, live cell imaging confocal microscopy pictures, and in vivo antitumor efficacy. Figure S1: Confocal microscopy images with conjugate **10** in the SSTR2 positive cell line AtT20 in absence (**left**) or 100-fold excess octreotide (**middle**), and in the SSTR2 negative A549 cell line (**right**), Figure S2: In vivo antitumor efficacy of conjugate **8** compared to vehicle and unconjugated mix of cryptophycin and octreotide (**left**), and body weight quantification during treatment (**right**), Video S1: Live cell imaging.
