# Supplementary material for: Octreotide Conjugates for Tumor Targeting and Imaging
Source: Pharmaceutics. 2019 May 7;11(5):220. doi: 10.3390/pharmaceutics11050220 (PMC6571972; doi:10.3390/pharmaceutics11050220)
Supplement: Supplementary file 1 [file pharmaceutics-11-00220-s001.zip › Octreotide conjugates for tumor targeting and imaging_SI.pdf]

Supporting information

for

Octreotide Conjugates for Tumor Targeting and Imaging

Eduard Figueras,[1,‡] Ana Martins,[1,2,‡] Adina Borbély,[1] Vadim Le Joncour,[3] Paola Cordella,[4] Raffaella Perego,[4] Daniela Modena,[4] Paolo Pagani,[4] Simone Esposito,[5] Giulio Auciello,[5] Marcel Frese,[1] Paola Gallinari,[2] Pirjo Laakkonen,[3] Christian Steinkühler,[2,4] and Norbert Sewald[1,*]

[1] Department of Chemistry, Organic and Bioorganic Chemistry, Bielefeld University, Universitätsstraße 25, DE-33615 Bielefeld, Germany

[2] Exiris srl, Via Via di Castel Romano 100, IT-00128 Rome, Italy

[3] Translational Cancer Medicine, Research Programs Unit, Haartmaninkatu 8, FI-00014 University Helsinki, Finland

[4] Italfarmaco SpA, Via dei Lavoratori 54, IT-20092 Cinisello Balsamo, Italy

[5] IRBM SpA, Via Pontina Km. 30,600, IT-00071 Pomezia, Italy

Author Information

‡ These authors contributed equally to this work

* Corresponding author

E-mail: norbert.sewald@uni-bielefeld.de

Table of contents

1) General methods 3

2) Synthesis of octreotide-Cy5.5 conjugate 7

3) Synthesis of PEG spacer 9

4) NMR, HPLC (λ = 220 nM) and mass spectra 11

5) Confocal microscopy 21

6) *In vivo* experiments 21

1) General methods

All reactions requiring anhydrous conditions were performed under argon atmosphere. DMF was dried over 4 Å molecular sieves, CH_2Cl_2 was distilled from CaH_2, THF was distilled from sodium/benzophenone. All the other chemicals and solvents (HPLC-grade or reagent-grade quality), unless otherwise stated, were purchased from commercial sources and used without further purification. Silica for flash chromatography was purchased from Macherey-Nagel 40-63 µM (230-400 mesh). Reactions were monitored by thin layer chromatography using aluminium-backed plates coated with silica gel 60 F254 from Merck; visualization was accomplished with UV light or staining with potassium permanganate or cerium molybdate solution.

High performance liquid chromatography - mass spectrometry

HPLC-MS was conducted using an Agilent 1200 series consisting of an autosampler, degasser, binary pump, column oven and diode array detector coupled to an Agilent 6220 accurate-mass TOF LC/MS. A Phenomenex Luna® 3 µm C18(2) 100 Å (100 mm x 2 mm) was used as column.

Eluent A: $H_2O/CH_3CN/HCO_2H$ = 95/5/0.1 and eluent B: $H_2O/CH_3CN/HCO_2H$ = 5/95/0.1.

Flow rate: 300 µL/min

0 min	100% A	0% B
10.0 min	2% A	98% B
11.0 min	2% A	98% B
11.5 min	100% A	0% B
15.0 min	100% A	0% B

High resolution mass spectrometry

High resolution mass spectra were recorded on an Agilent 6220 accurate-mass TOF LC/MS. Samples were injected through an Agilent 1200 series. Same solvents and column than HPLC-MS were used and a linear gradient from 0 to 98% B at 250 µL/min over 4 minutes was employed.

The mass spectrometer was externally calibrated using Agilent tuning mix prior to measurement.

Preparative reversed phase - high performance liquid chromatography

Preparative RP-HPLC was performed on a MERCK-HITACHI unit (controller: D-7000, pump: L7150, detector: L7420, UV-absorption measured at λ = 220 nm).

Eluent A: $H_2O/CH_3CN/TFA$ = 95/5/0.1 and eluent B: $H_2O/CH_3CN/TFA$ = 5/95/0.1

Method P1:

Column: Macherey-Nagel Nucleosil C_{18} (250 mm x 21 mm, 10 µm particle size)

Flow rate: 10 mL/min

0-2 min	100% A	0% B
35 min	0% A	100% B
40 min	0% A	100% B
45 min	100% A	0% B

Method P2:

Column: Macherey-Nagel Nucleosil C_{18} (250 mm x 21 mm, 10 µm particle size)

Flow rate: 10 mL/min

0-5 min	100% A	0% B
50 min	0% A	100% B
55 min	0% A	100% B
60 min	100% A	0% B

Method P3:

Column: Macherey-Nagel Nucleosil C_{18} (250 mm x 10 mm, 7 µm particle size)

Flow rate: 4 mL/min

0-15 min	100% A	0% B
60 min	0% A	100% B
65 min	0% A	100% B
70 min	100% A	0% B

Method P4:

Column: Macherey-Nagel Nucleosil C_{18} (250 mm x 10 mm, 7 µm particle size)

Flow rate: 4 mL/min

0-15 min	100% A	0% B
75 min	0% A	100% B
80 min	0% A	100% B
85 min	100% A	0% B

UPLC-HRMS conditions for plasma stability studies

Samples were analyzed on a system consisting of a Dionex Ultimate 3000 RS Pump coupled with (a) Dionex Ultimate 3000 RS from Thermo Scientific (Bremen, Germany) autosampler or (b) PAL LSI from CTC Analytics AG (Zwingen, Switzerland) autosampler. UPLC Peptide BEH C18 (50 mm x 2.1 mm, 1.7 µm, 130 Å) column from Waters (Wexford, Ireland) at 40 °C was used for chromatographic separation at a flow rate of 400 µL/min with a linear gradient composed of mobile phase A (0.1% formic acid in water) and B (0.1% formic acid in CH_3CN). A volume of (a) 2 µL or (b) 5 µL was injected.

Gradient:

0 min	99.5% A	0.5% B
4.0 min	5% A	95% B
5.0 min	5% A	95% B
5.1 min	99.5% A	0.5% B
6.0 min	99.5% A	0.5% B

All analyses were performed on a Q-Exactive Orbitrap™ mass spectrometer (Thermo Scientific) in ESI positive full scan/data-dependent MS/MS (FS-dd-MS/MS). Each cycle contains four scan events: Full Scan with *m/z* range (a) 150-1600 or (b) 200-2000 and resolution 35,000 FWHM at 200 *m/z*, mass accuracy: 5 ppm, followed by three MS/MS fragmentation scans with resolution 17,500 FWHM at 200 *m/z* over the three most abundant ions (Top N = 3) of the full-MS spectrum. The IS warfarin was detected in FS using the $[M+H]^+$ at *m/z*: 309.1121. Analysis of data was performed with XCalibur software. (a) was used for mouse plasma stability while (b) was used for human plasma stability.

HPLC-MS conditions for pharmacokinetic analysis

Samples were analyzed using a HPLC (Nexera, Shimadzu) connected to an Orbitrap Qexactive Focus (Thermo Scientific). A Jupiter C18 300 Å (50 mm x 2 mm) 5 µm particle size was used as column.

Eluent A: $H_2O/CH_3CN/HCO_2H$ = 90/10/0.1 and eluent B: CH_3CN/HCO_2H 99.9/0.1.
Flow rate: 400 µL/min

0 min	60% A	40% B
2.5 min	40% A	60% B
2.6 min	0% A	100% B
4.0 min	0% A	100% B
4.1 min	60% A	40% B
6.0 min	60% A	40% B

HPLC-MS conditions for cathepsin B cleavage studies

Samples were analyzed using a HPLC (Prominence, Shimadzu) connected to a triple quadrupole mass spectrometer (API4000, Sciex). A Jupiter C18 300 Å (50 mm x 2 mm) 5 µm particle size was used as column.

Eluent A: $H_2O/CH_3CN/HCO_2H$ = 90/10/0.1 and eluent B: ACN/HCO_2H 99.9/0.1.
Flow rate: 200 µL/min

0 min	60% A	40% B
5.0 min	60% A	40% B
5.1 min	0% A	100% B
7.0 min	0% A	100% B
7.1 min	60% A	40% B
10 min	60% A	40% B

NMR spectroscopy

NMR spectra were recorded on a Bruker Avance 500 or Avance 500HD spectrometer (^1H: 500 MHz) at 298 K. Chemical shifts were referenced to residual nondeuterated solvent signal ($CDCl_3$: ^1H: 7.26 ppm). Coupling constants (*J*) are reported in Hz with the following abbreviations used to indicate splitting: s = singlet, t = triplet, m = multiplet.

Photo spectrophotometer

Photo spectrometry was recorded in a UV-3100 PC from VWR®.

2) Synthesis of octreotide-Cy5.5 conjugate

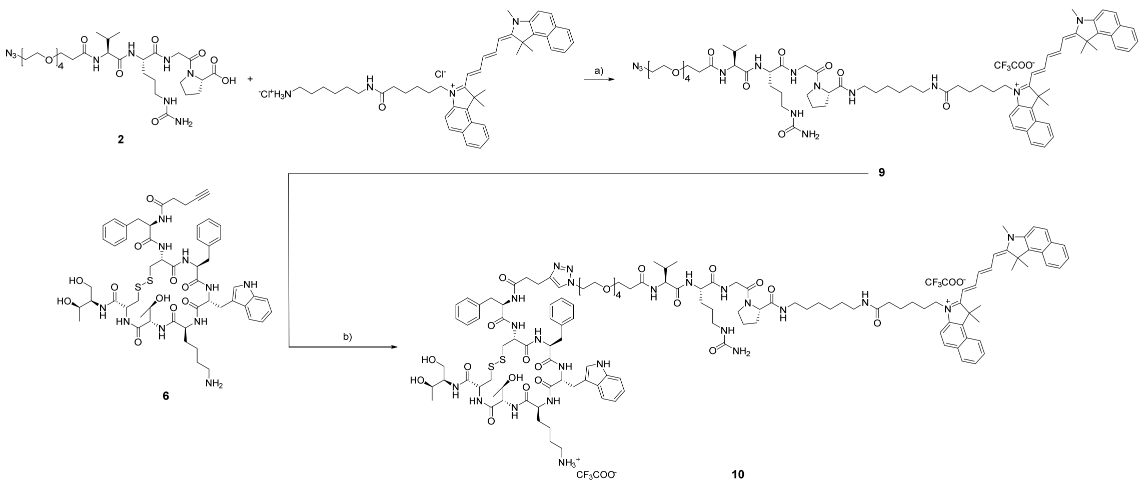

Scheme S1. Synthesis of octreotide-cy5.5 conjugate **10**. Reagents and conditions: a) PyBOP (2 eq), HOBt·H_2O (2.25 eq), DIPEA (2.5 eq), DMF, RT, 4 h; b) $CuSO_4 \cdot 5H_2O$ (0.6 eq), sodium ascorbate (0.4 eq), DMF/H_2O (1:1), 40 ºC, 24 h.

Synthesis of N_3-PEG4-Val-Cit-Gly-Pro-Cy5.5 (9):

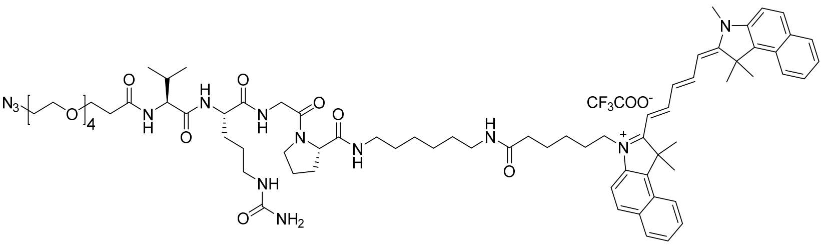

Cy5.5 (7.5 mg, 9.97 µmol, 1 eq), **5** (14 mg, 19.96 µmol, 2 eq), PyBOP (10.4 mg, 19.98 µmol, 2 eq) and HOBt·H_2O (3.4 mg; 22.2 µmol; 2.25 eq) were placed under argon atmosphere and dissolved with anhydrous DMF (0.5 mL). DIPEA (8.7 µL, 49.94 µmol, 5 eq) was added and the reaction mixture was stirred at RT for 4 h. Then, the solution was directly purified by RP-HPLC (method P3). Freeze-drying of fractions containing the product afforded **9** (14.4 mg, 98% yield) as blue powder.

HPLC-MS: t_R = 7.93 min, >99% purity (λ = 220 nm), *m/z* = 1364.91 (1364.81 $[M]^+$); 682.99 (682.91 $[M+H]^{2+}$)

HRMS (ESI-MS): *m/z* calculated for $C_{75}H_{107}N_{13}O_{11}$ $[M+H]^{2+}$ 682.9101; found 682.9130

Synthesis of 10:

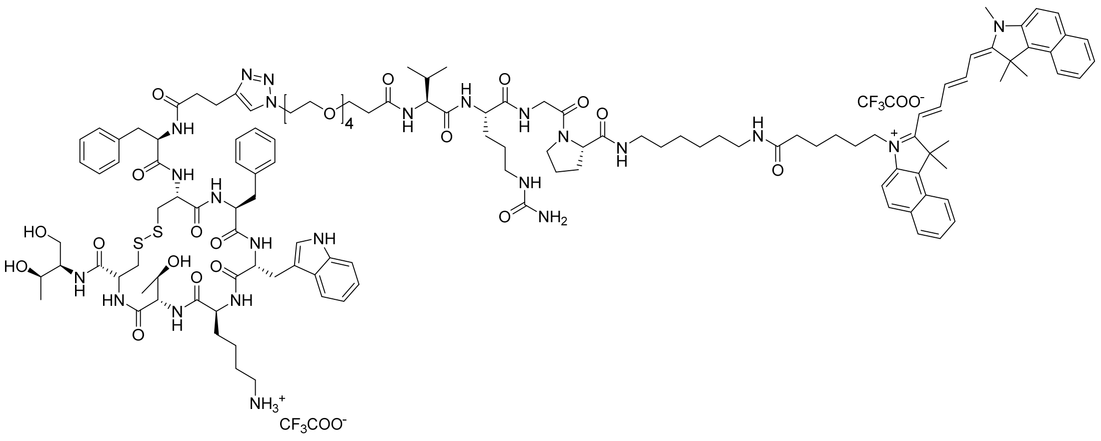

Azide **9** (4.5 mg, 3.05 µmol, 1 eq), alkyne **6** (4.1 mg, 3.38 µmol, 1.1 eq), $CuSO_4{\cdot}5H_2O$ (0.5 mg, 0.6 eq) and sodium ascorbate (0.24 mg, 0.4 eq) were placed under argon atmosphere and dissolved with a degassed solution of DMF/H_2O (1:1, 0.5 mL). The solution was stirred for 24 h at 40 ºC and was directly purified by RP-HPLC (method P3). Freeze-drying of desired fractions afforded **10** (6 mg, 73% yield) as blue powder.

HPLC-MS: t_R = 6.99 min, >99% purity (λ = 220 nm), *m/z* = 1232.22 (1232.14 $[M+H]^{2+}$); 821.82 (821.76 $[M+2H]^{3+}$); 616.62 (616.58 $[M+3H]^{4+}$)

HRMS (ESI-MS): *m/z* calculated for $C_{129}H_{178}N_{23}O_{22}S_2$ $[M+2H]^{3+}$ 821.7647; found 821.7670; calculated for $C_{129}H_{179}N_{23}O_{22}S_2$ $[M+3H]^{4+}$ 616.5754; found 616.5768

3) Synthesis of PEG spacer

***tert*-Butyl-15-hydroxy-4,7,10,13-tetraoxapentadecanoate (11):**

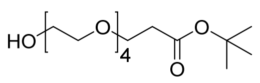

To a solution of tetraethyleneglycol (40.61 mL, 45.64 g, 235 mmol) in anhydrous THF (125 mL) a piece of sodium (1/4 cm) was added. After the sodium had reacted completely, *tert*-butylacrylate (11.98 mL, 10.57 g, 82.5 mmol) was added dropwise over 20 min and the resulting solution was stirred at RT overnight. The pH was adjusted to 7-8 with NaOH solution (1 N) and the solvents were removed in vacuum. The residue was dissolved in sat. NaCl solution (75 mL) and extracted with EtOAc (3 x 100 mL). The combined organic layers were dried over $MgSO_4$ and the solvent was removed under reduced pressure to obtain **11** (21.87 g, 82% yield) as colorless oil.

^{1}H-NMR (500 MHz, $CDCl_3$): δ (ppm) = 1.44 (s, 9H, $C(CH_3)_3$), 2.50 (t, *J* = 6.6 Hz, 2H, CH_2COO^tBu), 3.59-3.73 (m, 18H, OCH_2).

***tert*-Butyl 15-Azido-4,7,10,13-tetraoxapentadecanoate (12):**

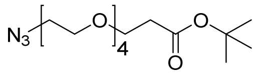

11 (1.50 g, 4.65 mmol, 1 eq) was dissolved in anhydrous THF (10 mL) and the resulting solution was cooled to 0°C. Methanesulfonyl chloride (0.54 mL, 6.98 mmol, 1.5 eq) and triethylamine (0.97 mL, 6.98 mmol, 1.5 eq) were added dropwise and the solution was stirred for 30 min at 0 °C and then overnight at RT. $NaHCO_3$ (0.27 g, 3.20 mmol, 0.7 eq) and NaN_3 (0.45 g, 6.98 mmol, 1.5 eq) were added with dist. water (10.5 mL) to the mixture and the resulting solution was stirred for 20 min at RT. THF was removed in vacuum and the remaining solution was stirred at 80 °C for 4 h. After cooling down, the mixture was extracted with DCM (3 x 30 mL), the combined organic layers were dried over $MgSO_4$ and the solvent was removed under reduced pressure. The crude was purified by column chromatography using PE/EtOAc (1:1) as eluent to obtain **12** (0.74 g; 46% yield) as colorless oil.

^{1}H-NMR (500 MHz, $CDCl_3$): δ (ppm) = 1.44 (s, 9H, $C(CH_3)_3$), 2.49 (t, 2H, *J* = 6.6 Hz, CH_2COO^tBu), 3.38 (t, 2H, *J* = 5.1 Hz, CH_2N_3), 3.59-3.68 (m, 14H), 3.70 (t, 2H, *J* = 6.6 Hz, $C\underline{H_2}CH_2COO^tBu$).

15-Azido-4,7,10,13-tetraoxapentadecanoic acid (13):

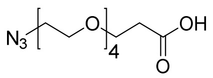

12 (0.22 g; 0.63 mmol) was dissolved in dry DCM (5 mL) and H_2O (0.25 mL). Then, TFA (5 mL) was added and the solution was stirred at RT for 1.5 h. The solvents were removed under reduced pressure and the product was coevaporated with diethyl ether (2 x 10 mL) to obtain **13** (0.18 g, 98% yield) as colorless oil.

^{1}H-NMR (500 MHz, $CDCl_3$): δ (ppm) = 2.65 (t, 2H, *J* = 6.1 Hz, CH_2COOH), 3.40 (t, 2H, *J* = 5.0 Hz, CH_2N_3), 3.64-3.70 (m, 14H), 3.78 (t, 2H, *J* = 6.1 Hz, C<u>H2</u>CH_2COOH).

4) NMR, HPLC (λ = 220 nM) and mass spectra

1: HPLC-MS

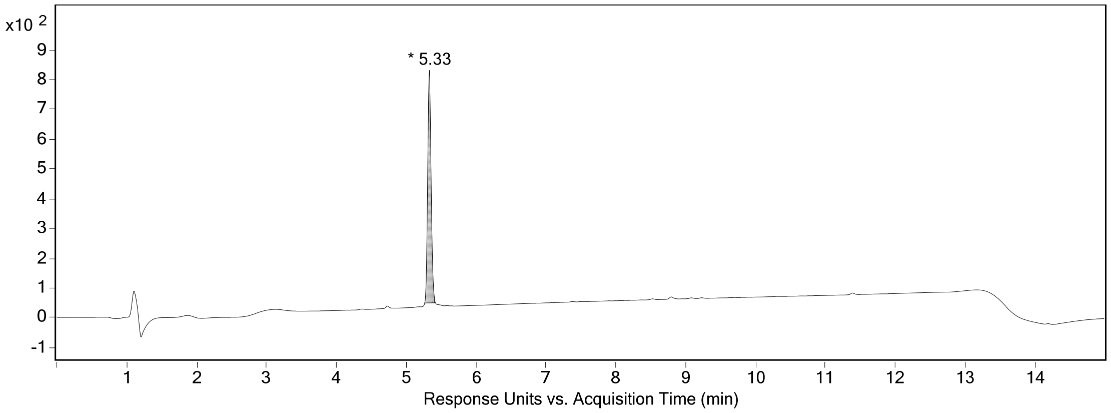

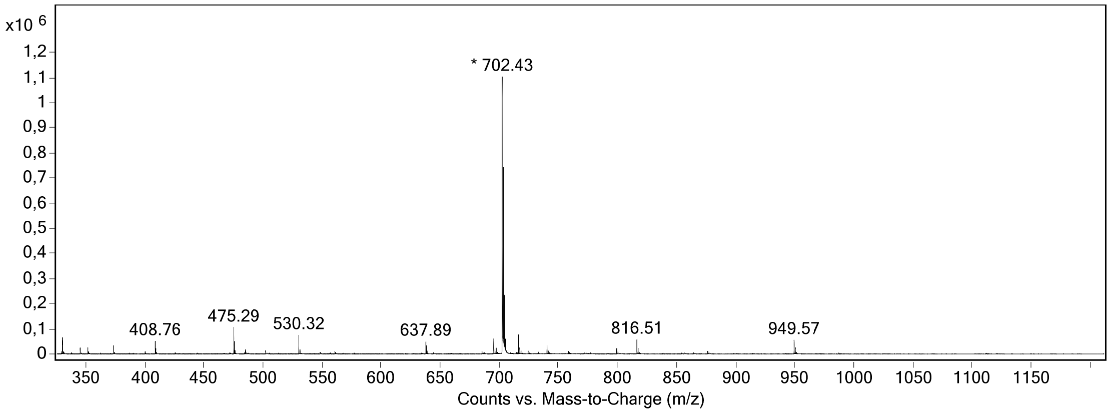

2: HPLC-MS

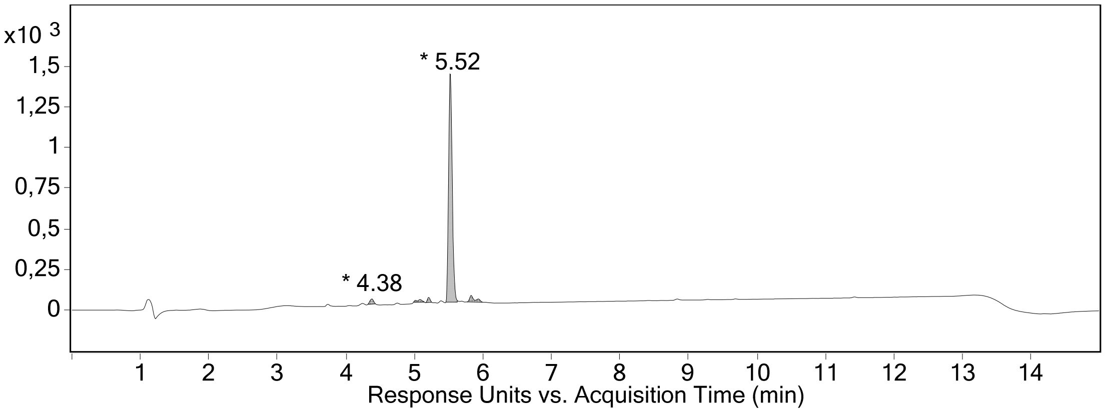

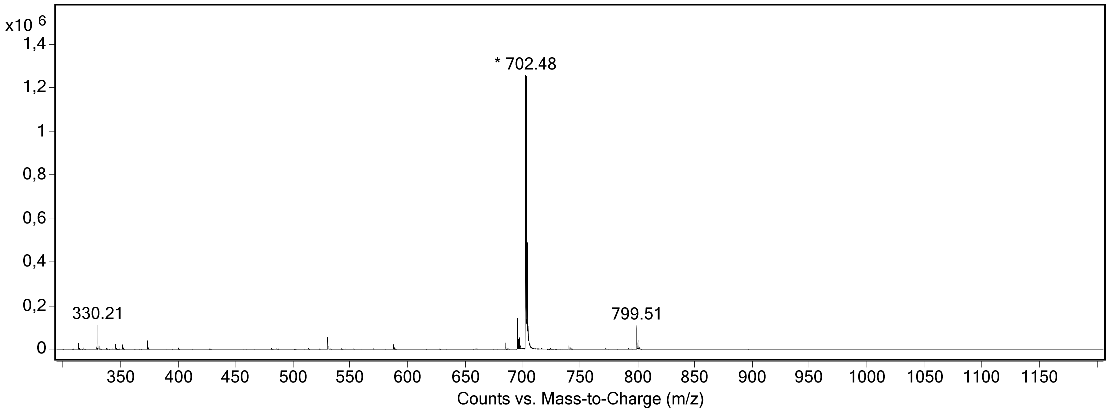

4: HPLC-MS and HRMS

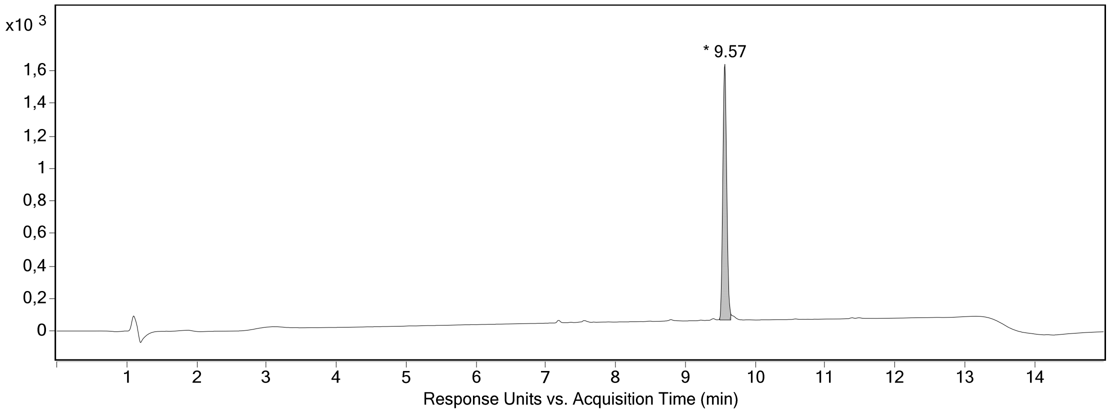

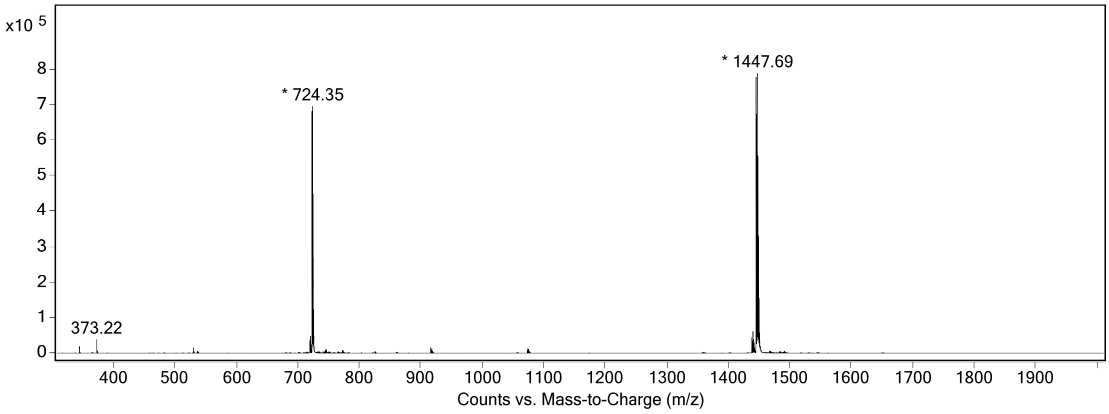

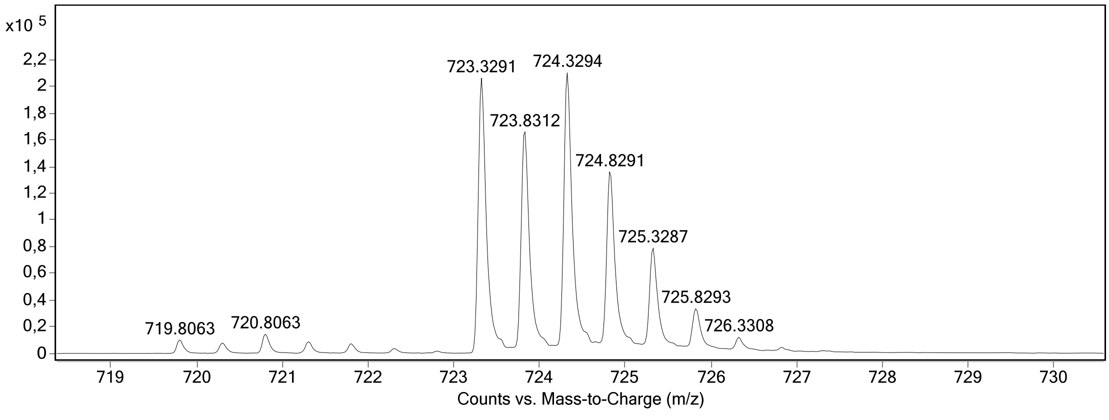

5: HPLC-MS and HRMS

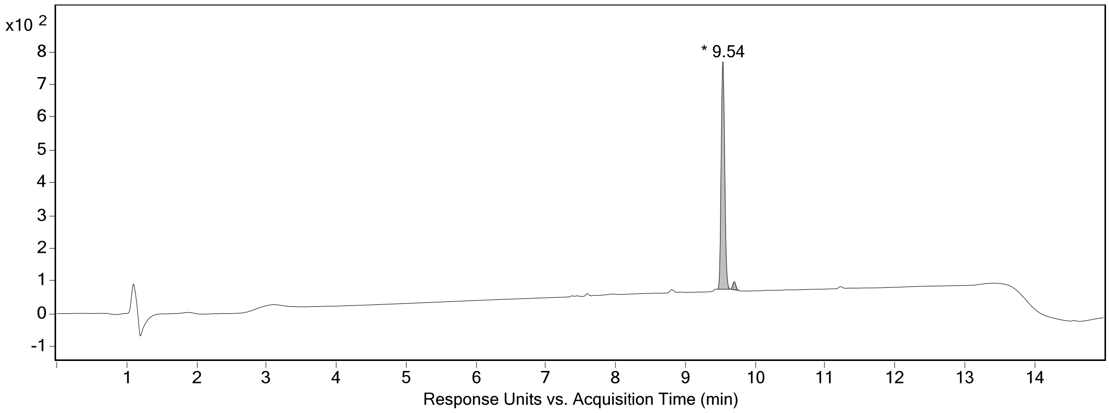

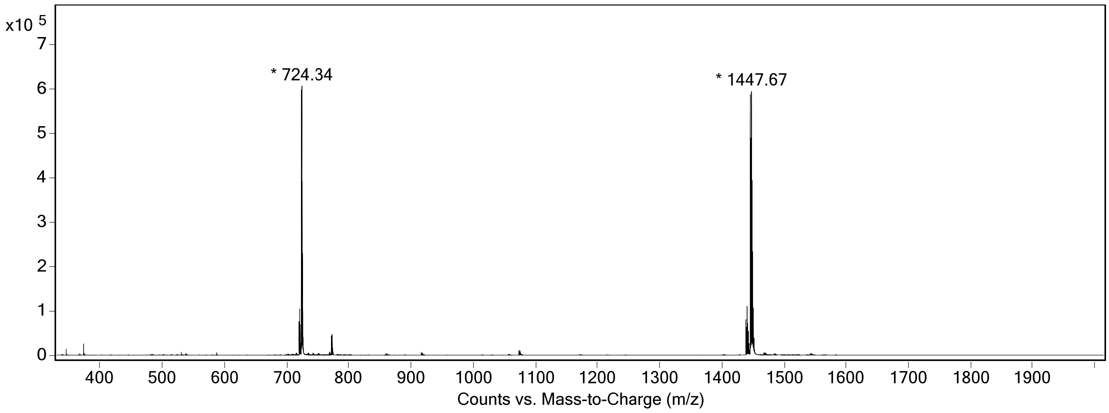

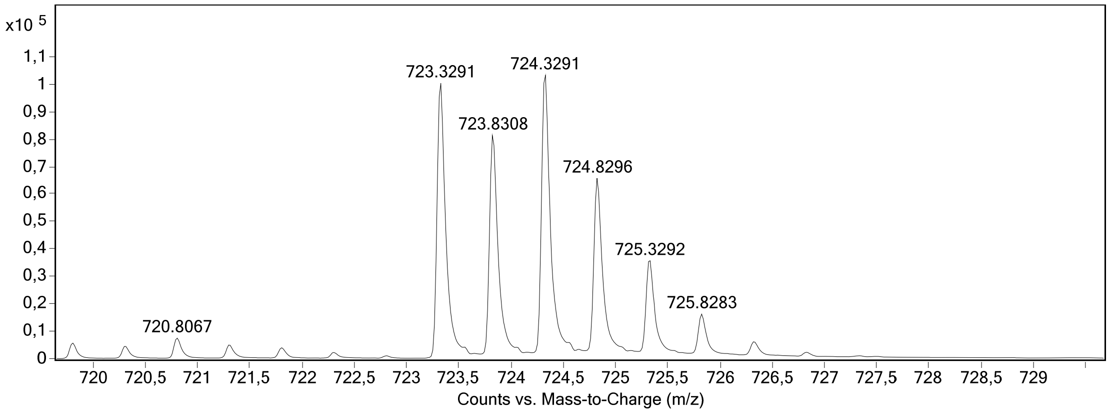

6: HPLC-MS and HRMS

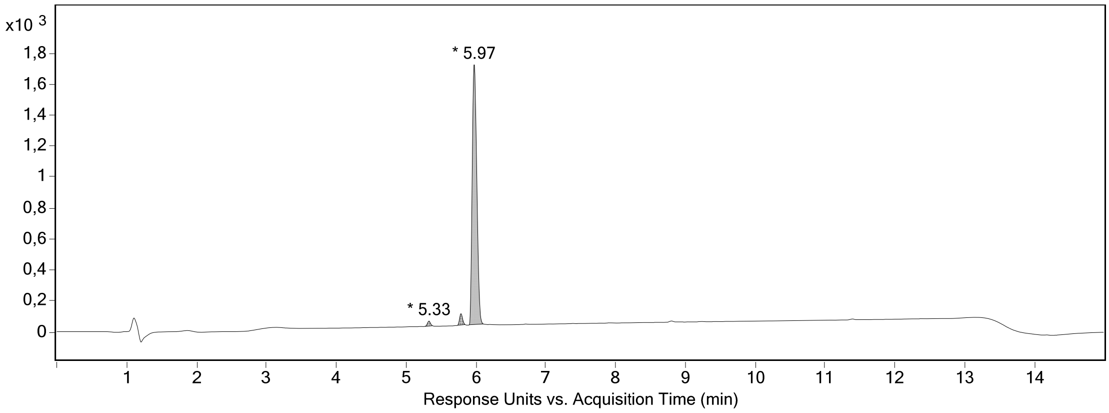

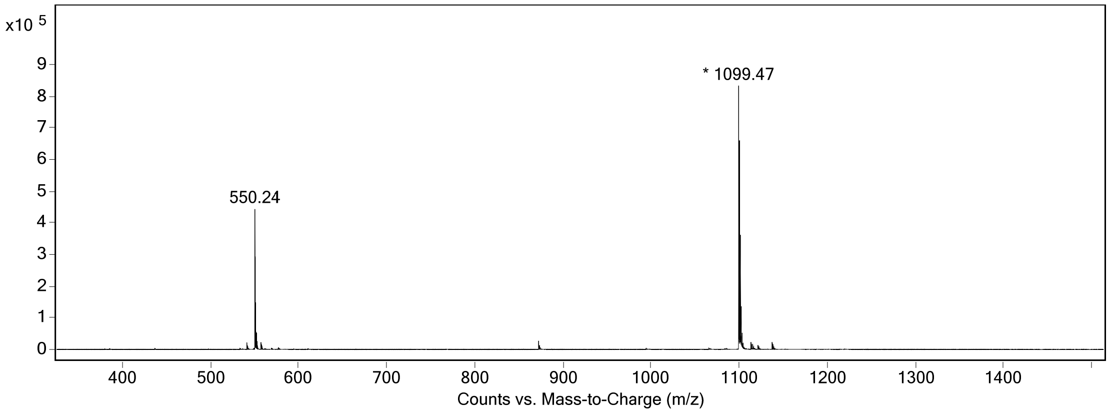

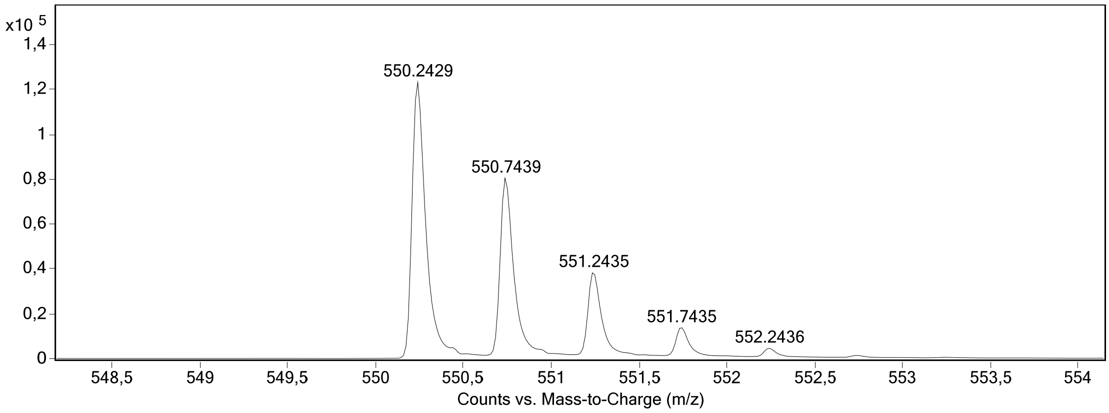

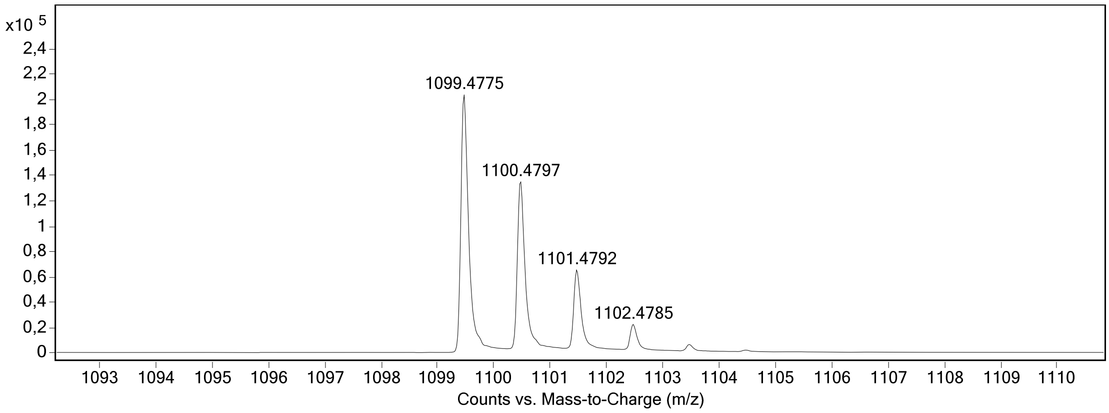

7: HPLC-MS and HRMS

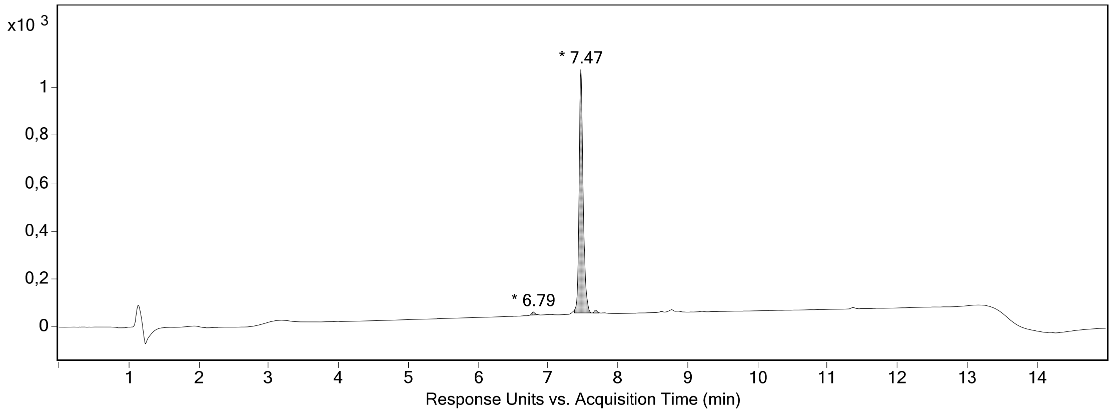

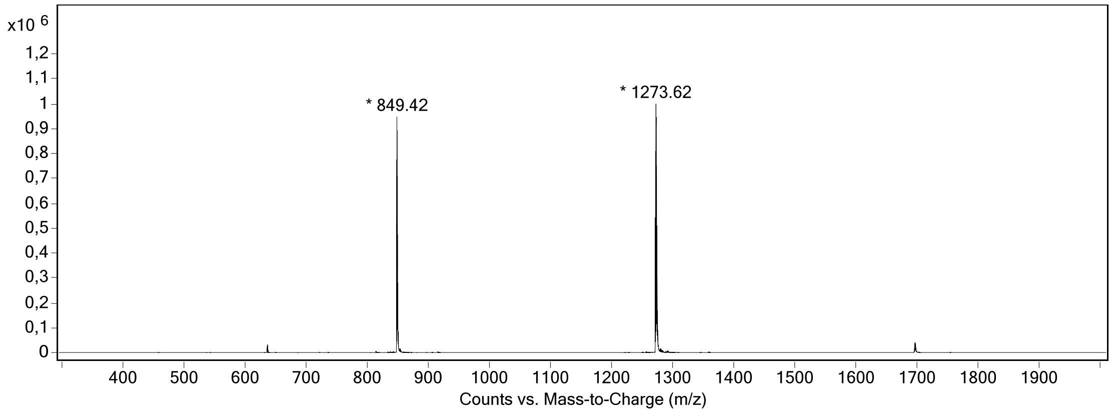

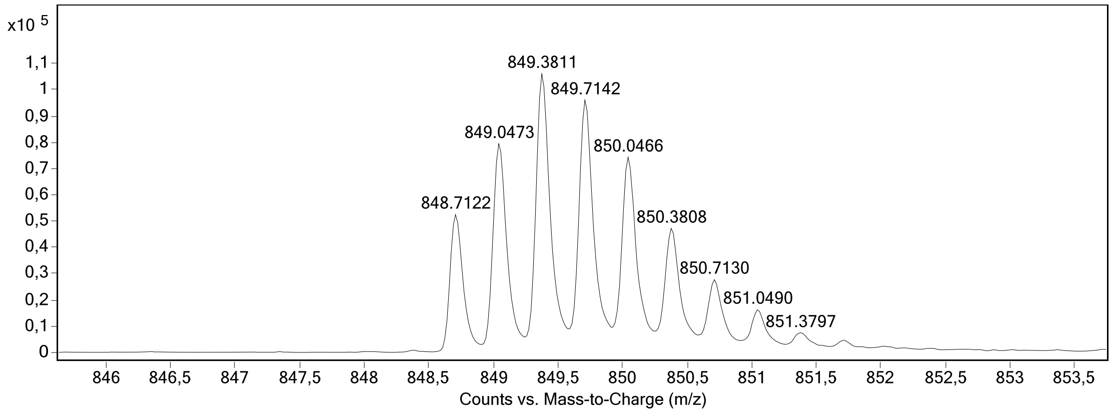

8: HPLC-MS and HRMS

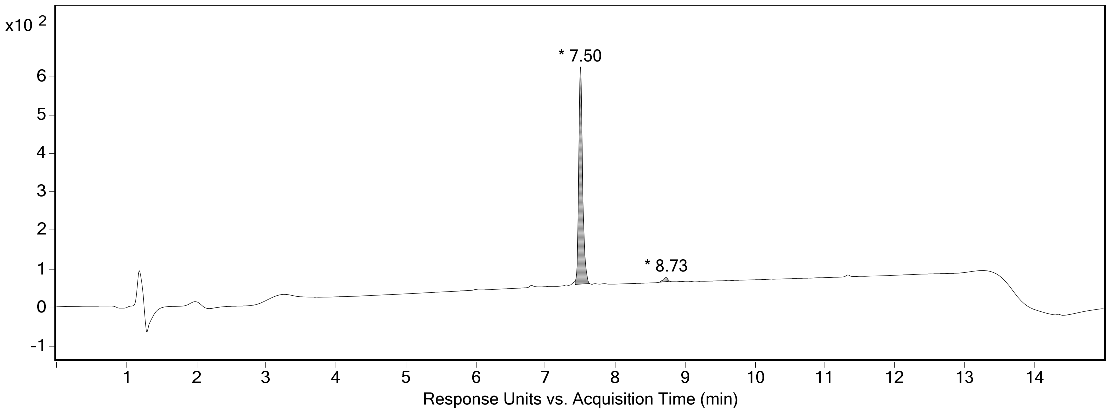

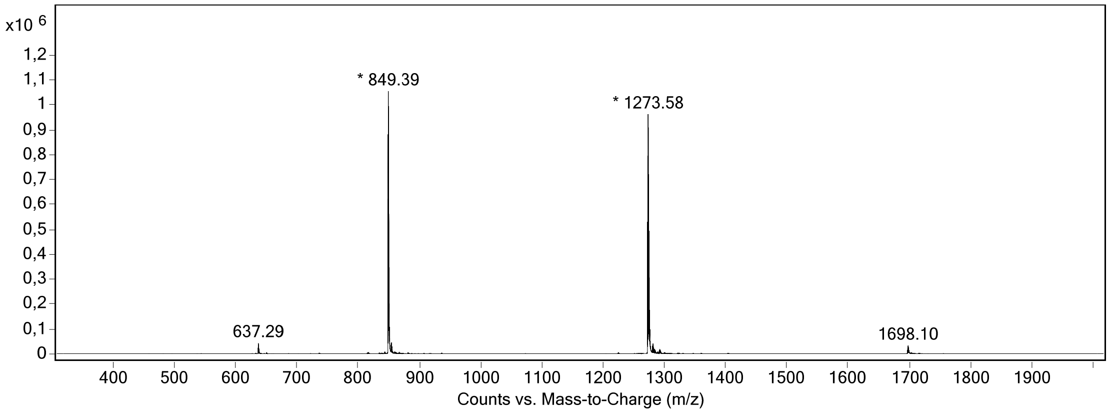

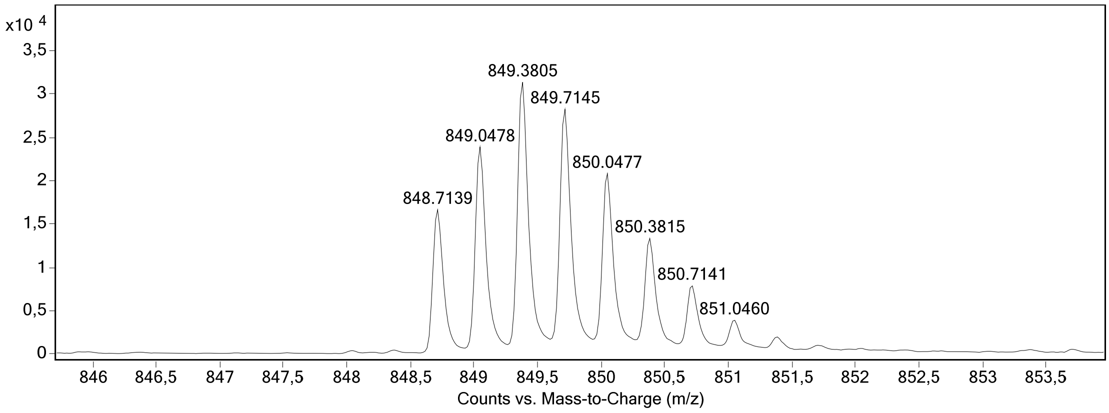

9: HPLC-MS and HRMS

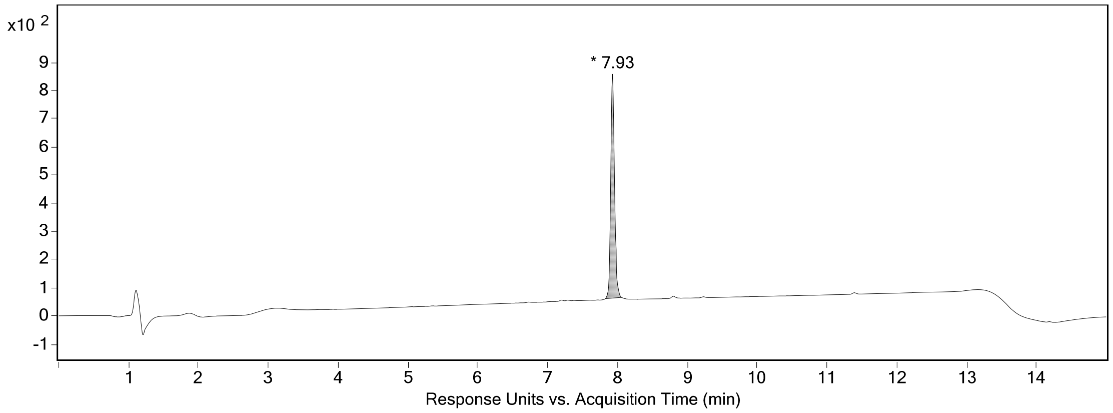

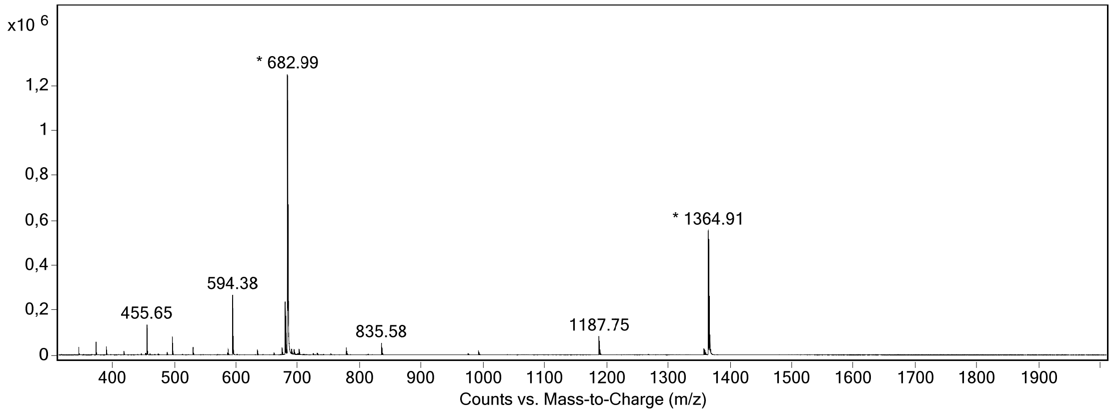

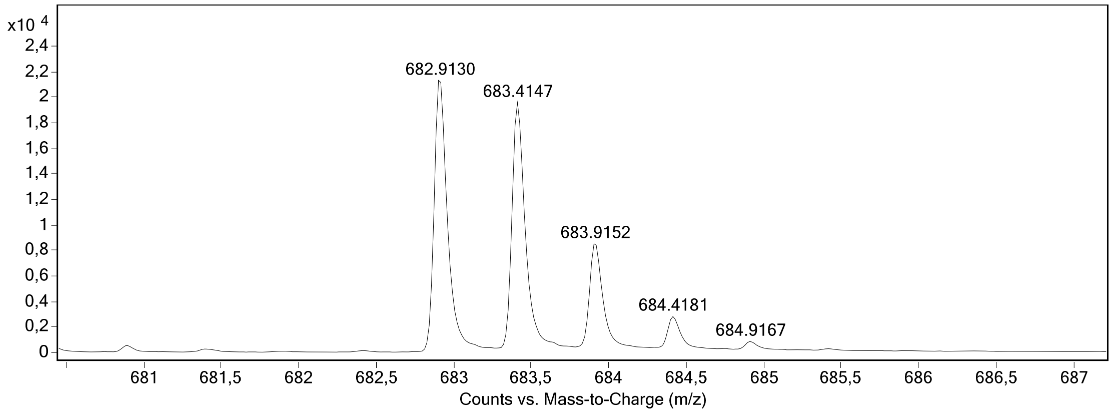

10: HPLC-MS and HRMS

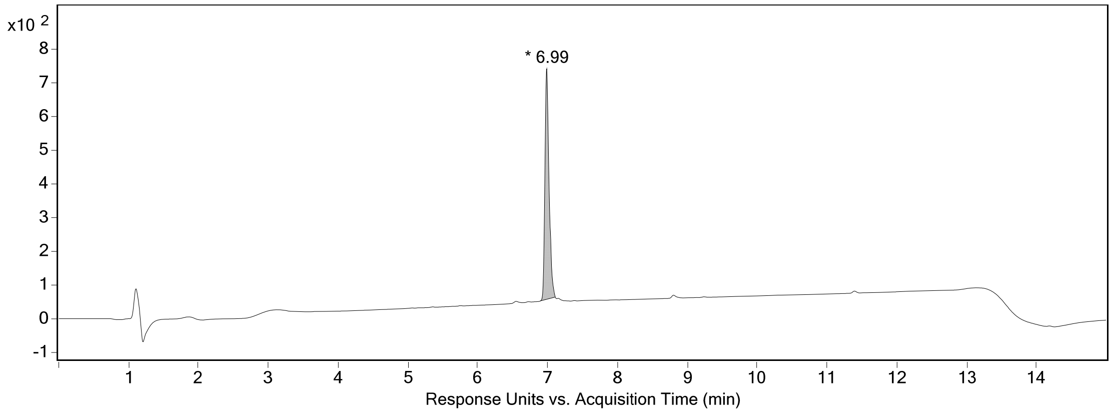

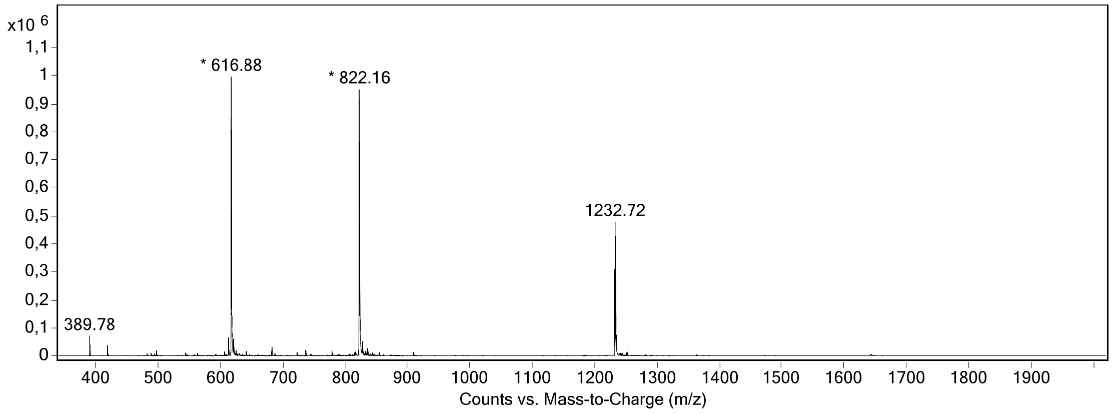

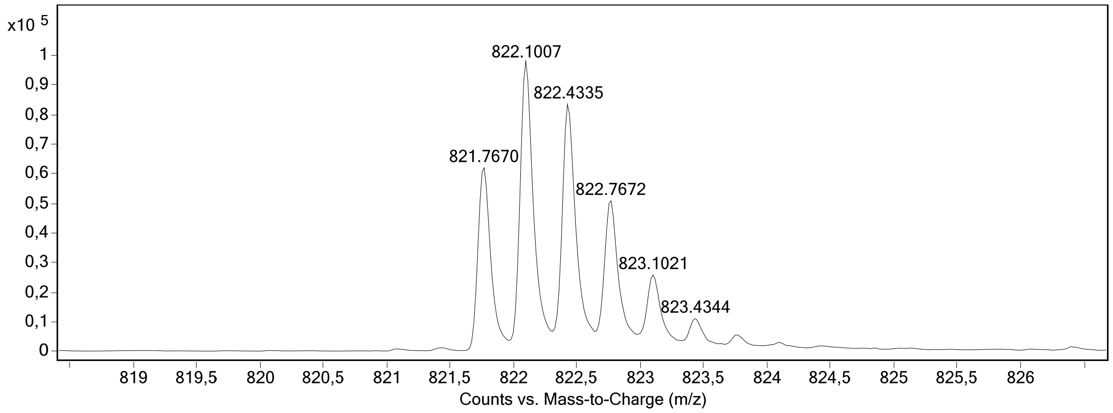

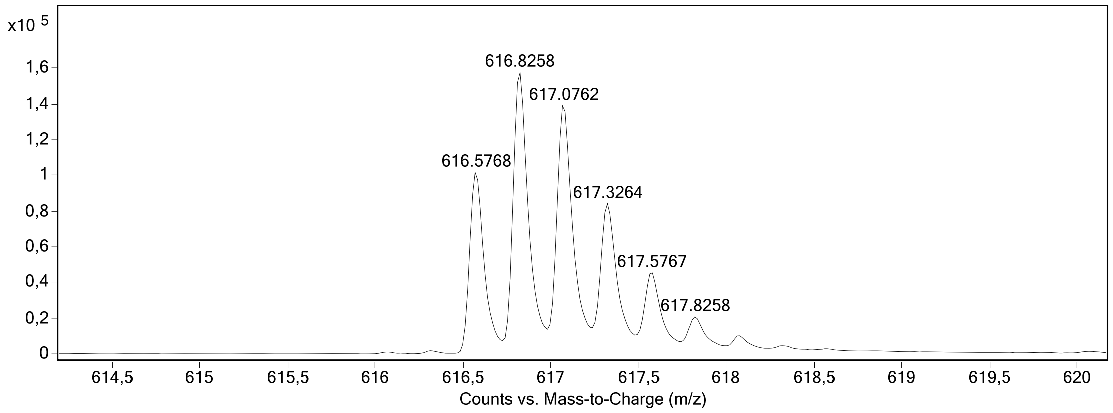

11: ^{1}H-NMR

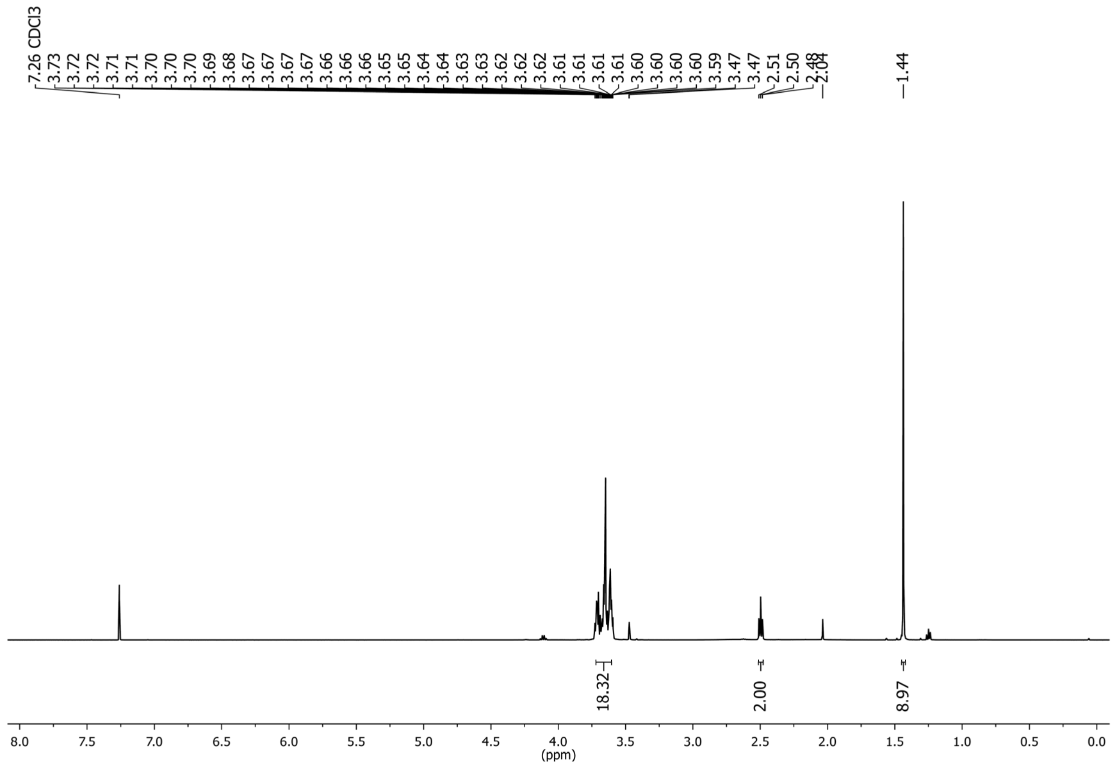

12: ^{1}H-NMR

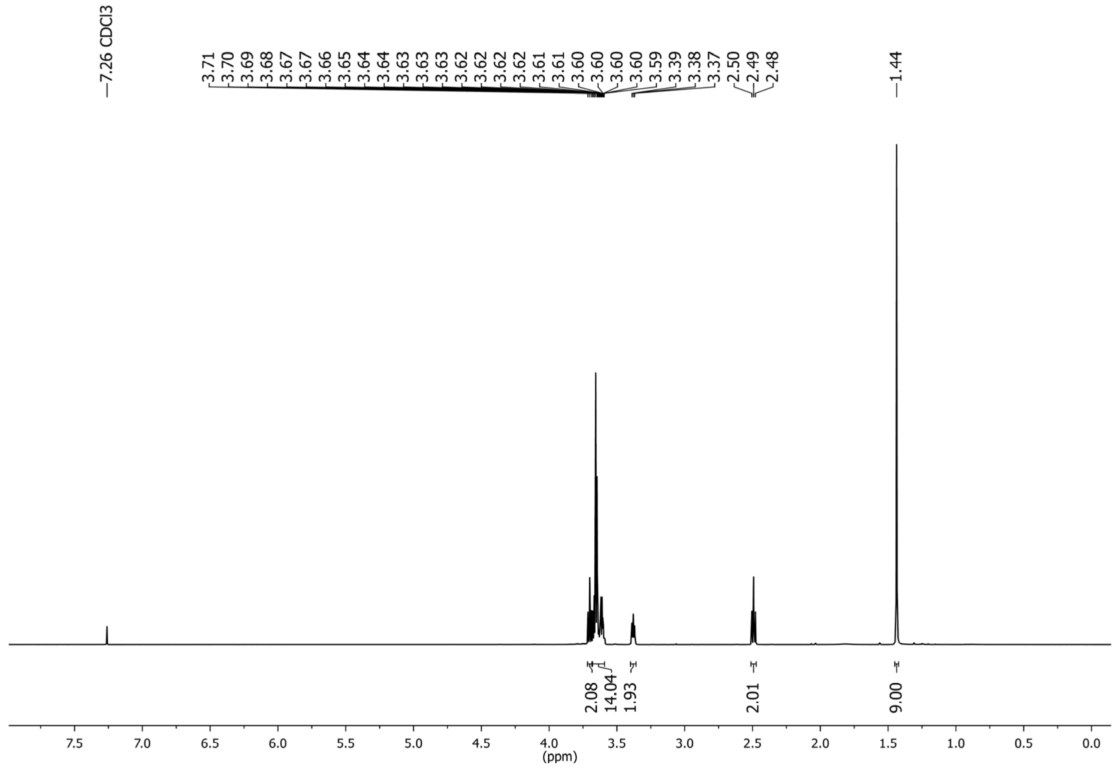

13: ^{1}H-NMR

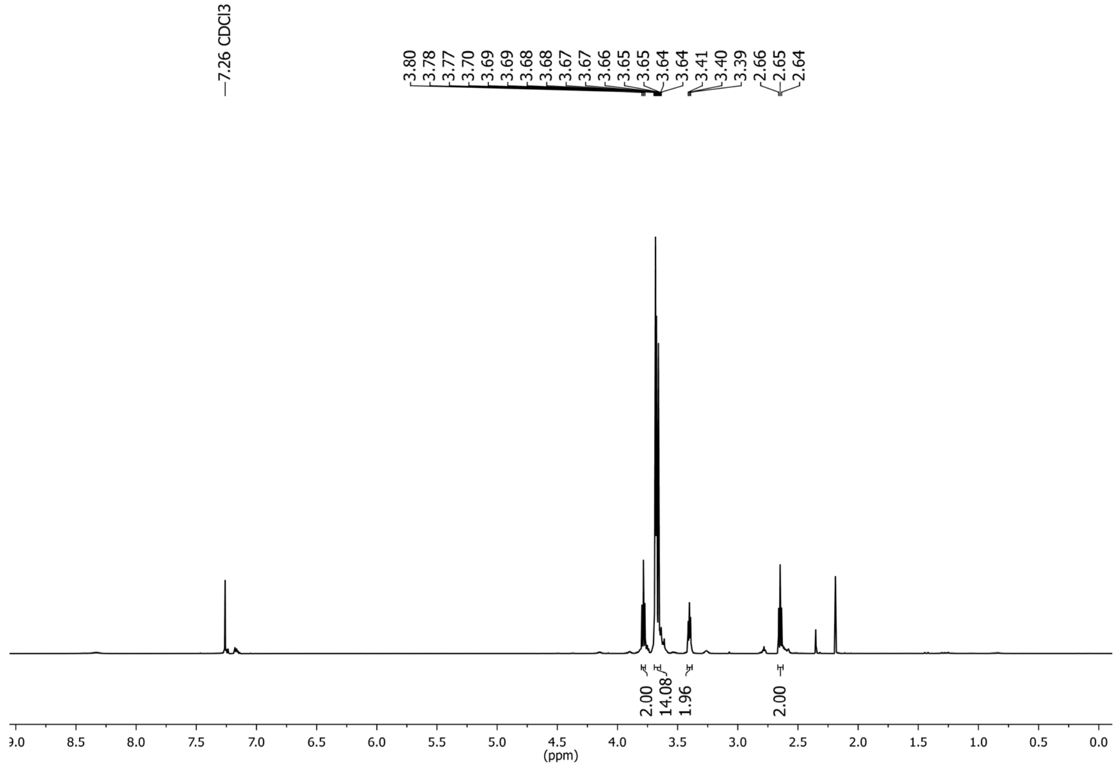

7.26 CDCl3
3.80
3.78
3.77
3.70
3.69
3.69
3.68
3.68
3.67
3.67
3.66
3.65
3.65
3.64
3.64
3.41
3.40
3.39
2.66
2.65
2.64
2.00
14.08
1.96
2.00
(ppm)

5) Confocal microscopy

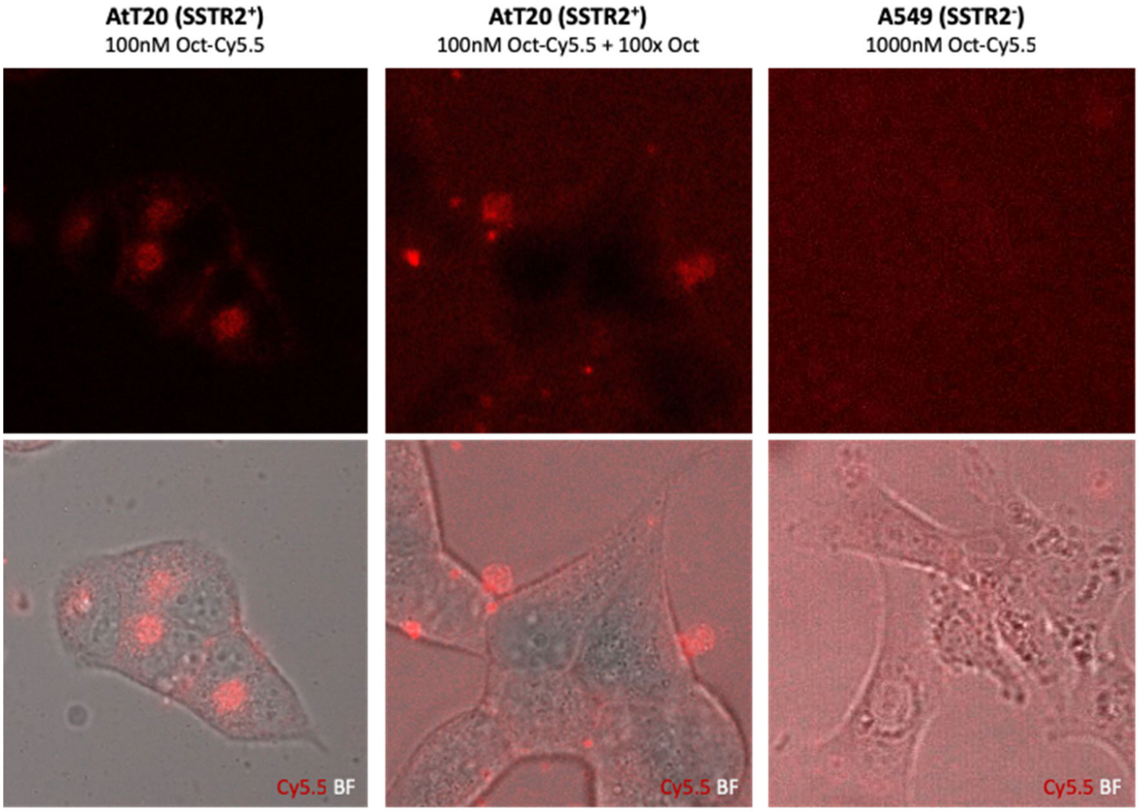

Figure S1. Confocal microscopy images from conjugate **10** in SSTR2 positive cell line AtT20 in absence (left) or 100-fold excess octreotide (middle), and in SSTR2 negative A549 cell line (right).

6) *In vivo* experiments

Animal experiments were approved by the Committee for Animal Experiments of the District of Southern Finland (ESAVI/6285/04.10.07/2014).

Implantation of tumors

For *in vivo* tumor targeting and antitumor efficacy experiments, three million AtT20/D16v-F2 murine pituitary tumor cells in 100 µL PBS/Matrigel (v/v) were xenografted in the right flank of four-weeks-old female BALB/c nude mice (BALB/cAnNRj-Foxn1$^{nu/nu}$, Janvier Labs). The experiments started around 15 days after implantation, when the animals started to show physical signs of the hormone releasing tumor.

In vivo antitumor efficacy

Animals bearing AtT20 tumors were randomized in three treatment groups (*n*=9) and injected intravenously with 5 mg/kg of conjugate **8** in 2% DMSO in water or a mix of unconjugated octreotide and cryptophycin in the respective molar concentration. Treatment was given once a week for a total of three weeks. Vehicle treatment was used as control. Mice were weighed, and the tumor sizes were monitored with an electronic caliper. Tumor volume was calculated using the ellipsoid formula $\nu = \pi/6 \cdot x \cdot y \cdot z$.

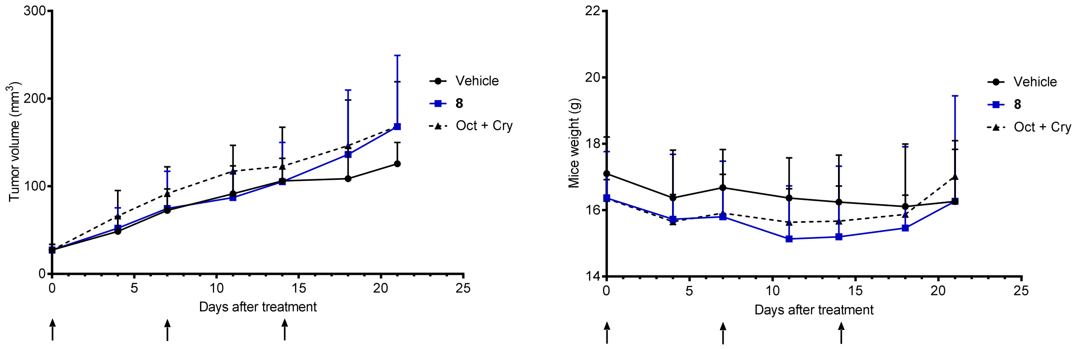

Figure S2. *In vivo* antitumor efficacy of conjugate **8** compared to vehicle and unconjugated mix of cryptophycin and octreotide (left), and body weight quantification during treatment (right).
